# Supplementary material for: Arginine methylation-dependent METTL14-SMN interaction regulates RNA m6A homeostasis
Source: EMBO Rep. 2025 Oct 6;26(22):5483–500. doi: 10.1038/s44319-025-00590-7 (PMC12635257; doi:10.1038/s44319-025-00590-7)
Supplement: Supplementary file 1 — Appendix [file 44319_2025_590_MOESM1_ESM.pdf]

Appendix: Table of Contents

Appendix Figure S1	2 – 3
Appendix Figure S2	4
Appendix Figure S3	5 – 6
Appendix Figure S4	7 – 8
Appendix Figure S5	9 – 10
Appendix Figure S6	11 – 12
Appendix Figure S7	13 – 14
Appendix Table S1	15 – 16

Appendix Figure S1

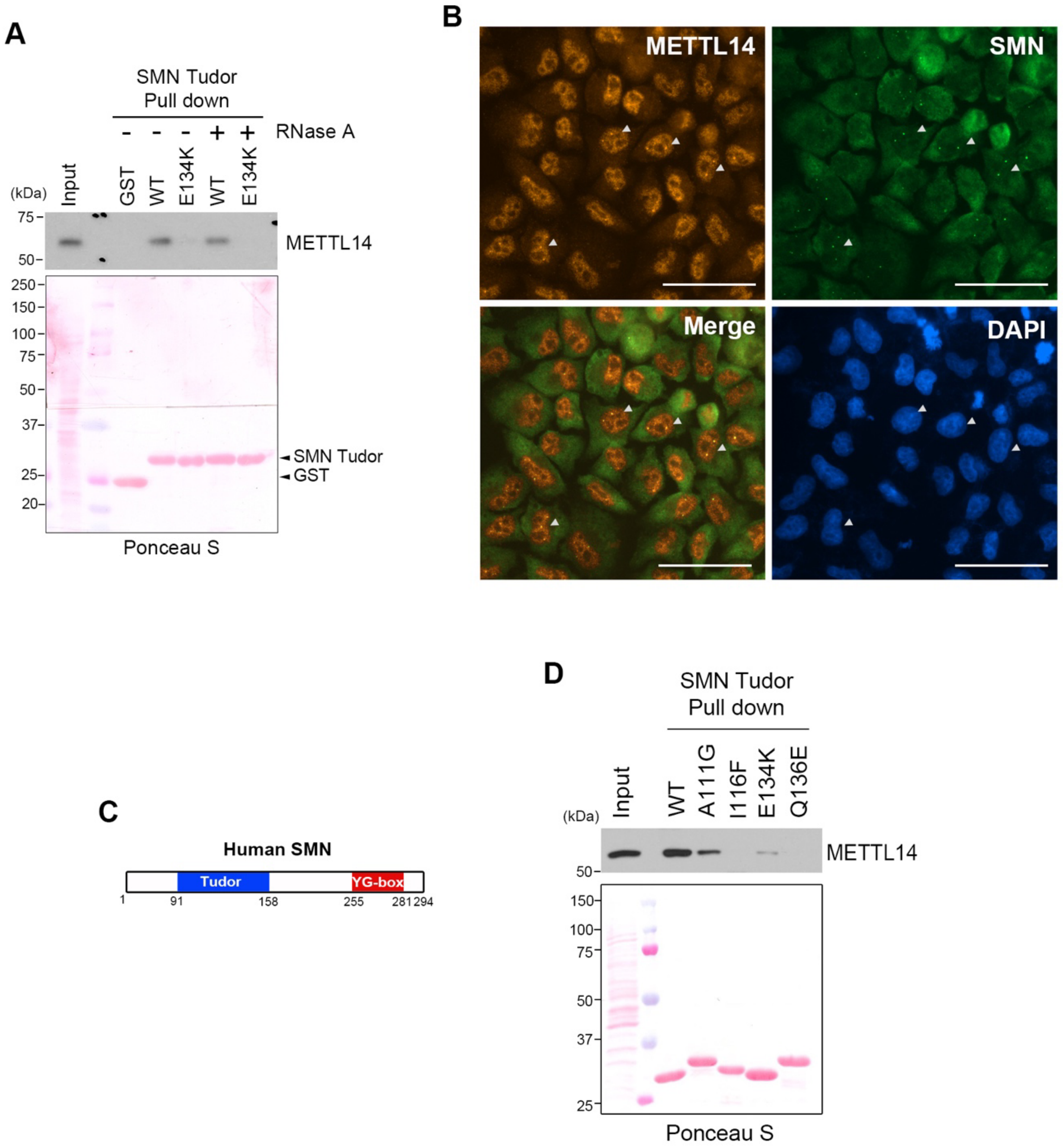

Appendix Figure S1. SMN interacts with METTL14.

- **(A)** The SMN-METTL14 interaction is not mediated by RNA. GST pull-down of SMN Tudor domain with HeLa cell lysates were performed in the presence of RNase A to disrupt potential RNA-mediated interaction.
- **(B)** METTL14 partially colocalizes with SMN in cells. Immunofluorescence was performed to detect the subcellular localizations of METTL14 and SMN in HeLa cells. White arrows indicate the cells in which METTL14 partially colocalizes with SMN, likely in Cajal bodies. Scale bar indicates 100 μM.
- **(C)** A diagram demonstrates the domain structure of the human SMN protein.
- **(D)** SMA patient-derived mutations that are located in the Tudor domain dampened the Tudor domain interaction with METTL14. GST pull-down was performed by incubating recombinant WT and various mutant Tudor domains of SMN with HeLa cell lysates.

Appendix Figure S2

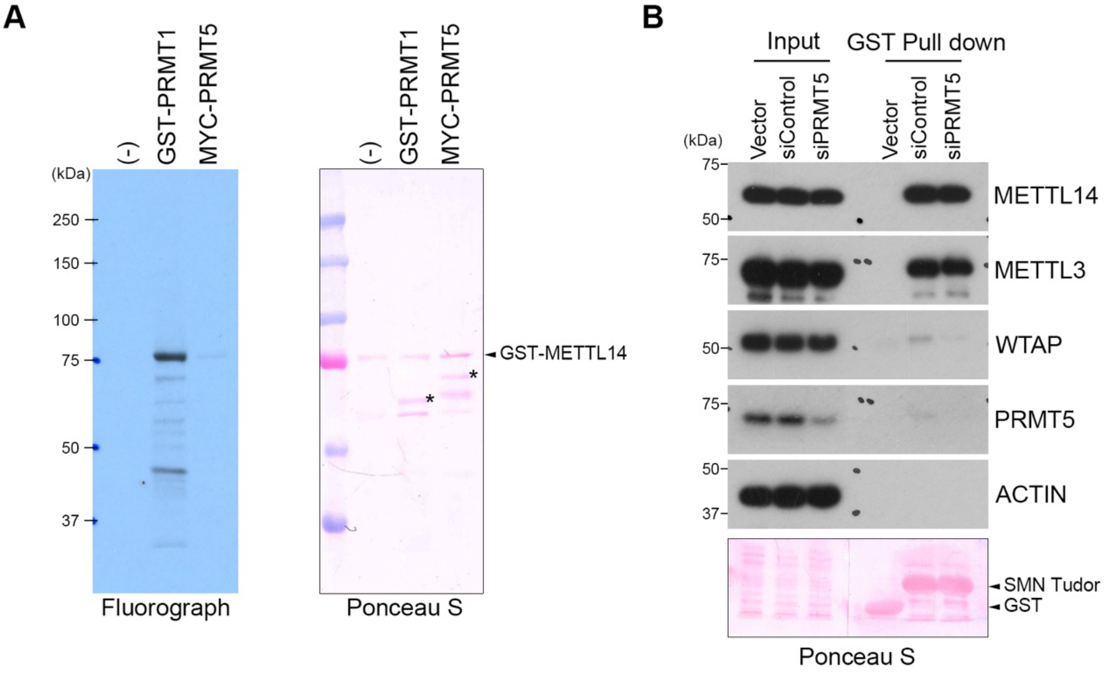

Appendix Figure S2. PRMT5 only weakly methylates METTL14 in vitro and marginally affects METTL14 interaction with SMN in cells.

- **(A)** PRMT5 exhibits much weaker methylation activity on METTL14 in comparison to PRMT1. In vitro arginine methylation was performed by incubating recombinant METTL14 with either PRMT1 (GST-PRMT1 from bacteria) or PRMT5 (Myc-PRMT5 from HEK293 cells). Black arrow indicates GST-METTL14 recombinant protein; asterisks indicate the enzymes.
- **(B)** Knockdown PRMT5 has a marginal effect on METTL14 interaction with SMN. GST pull-down assay was performed by incubating recombinant Tudor domain of SMN with HeLa cell lysates transfected with control siRNA (siControl) and PRMT5-specific siRNA (siPRMT5).

Appendix Figure S3

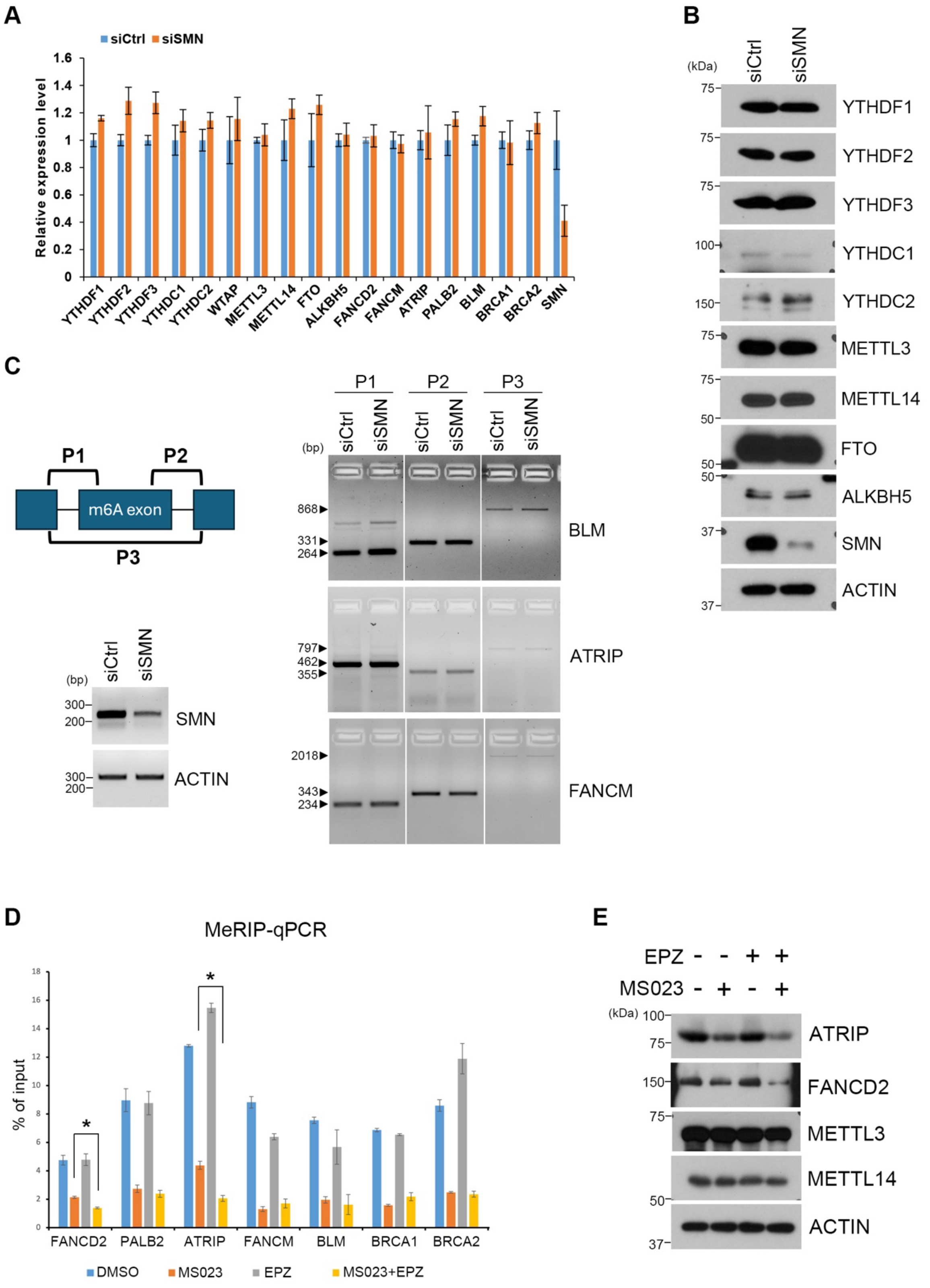

Appendix Figure S3. Knockdown of SMN or inhibition of PRMT activity reduces m^6A deposition and protein expression of DNA repair genes.

- **(A)** Knockdown of SMN does not affect the mRNA expression levels of m^6A “writers”, “readers”, and “erasers”. The expression levels of DNA repair genes are also largely unchanged. RT-qPCR was performed to detect gene expression in control (siControl) and SMN knockdown (siSMN) HeLa cells. Data are shown as mean ± SD from three biological replicates.
- **(B)** Knockdown of SMN does not affect the overall protein expression levels of m^6A “writers”, “readers”, and “erasers”. Western blot was performed with total cell lysates from control (siControl) and SMN knockdown (siSMN) HeLa cells.
- **(C)** Knockdown SMN does not affect the splicing of m^6A-containing exons. A diagram was shown to indicate three PCR products designed to measure the proper incorporation of m6A-containing exons. RT-PCR was performed using indicated primer pairs on RNA samples extracted from HeLa cells transfected with control siRNA (siCtrl) and SMN-specific siRNA (siSMN). No exon skipping products were detected with the three genes being tested.
- **(D)** Both type I and type II PRMT’s activity contribute to the regulation of m^6A deposition on DNA repair genes, specifically FANCD2 and ATRIP. MeRIP-qPCR was performed using RNA samples from HeLa cells treated with DMSO, MS023, EPZ, and MS023 plus EPZ to quantitatively measure the levels of m^6A on selected DNA repair genes. Data are shown as mean ± SD from three biological replicates. *, $p < 0.05$.
- **(E)** Both type I and type II PRMT’s activity contribute to the regulation of DNA repair gene expression. The levels of protein expression on ATRIP and FANCD2 were detected by Western blot on total cell lysates of HeLa cells treated with DMSO, MS023, EPZ, and MS023 plus EPZ.

Appendix Figure S4

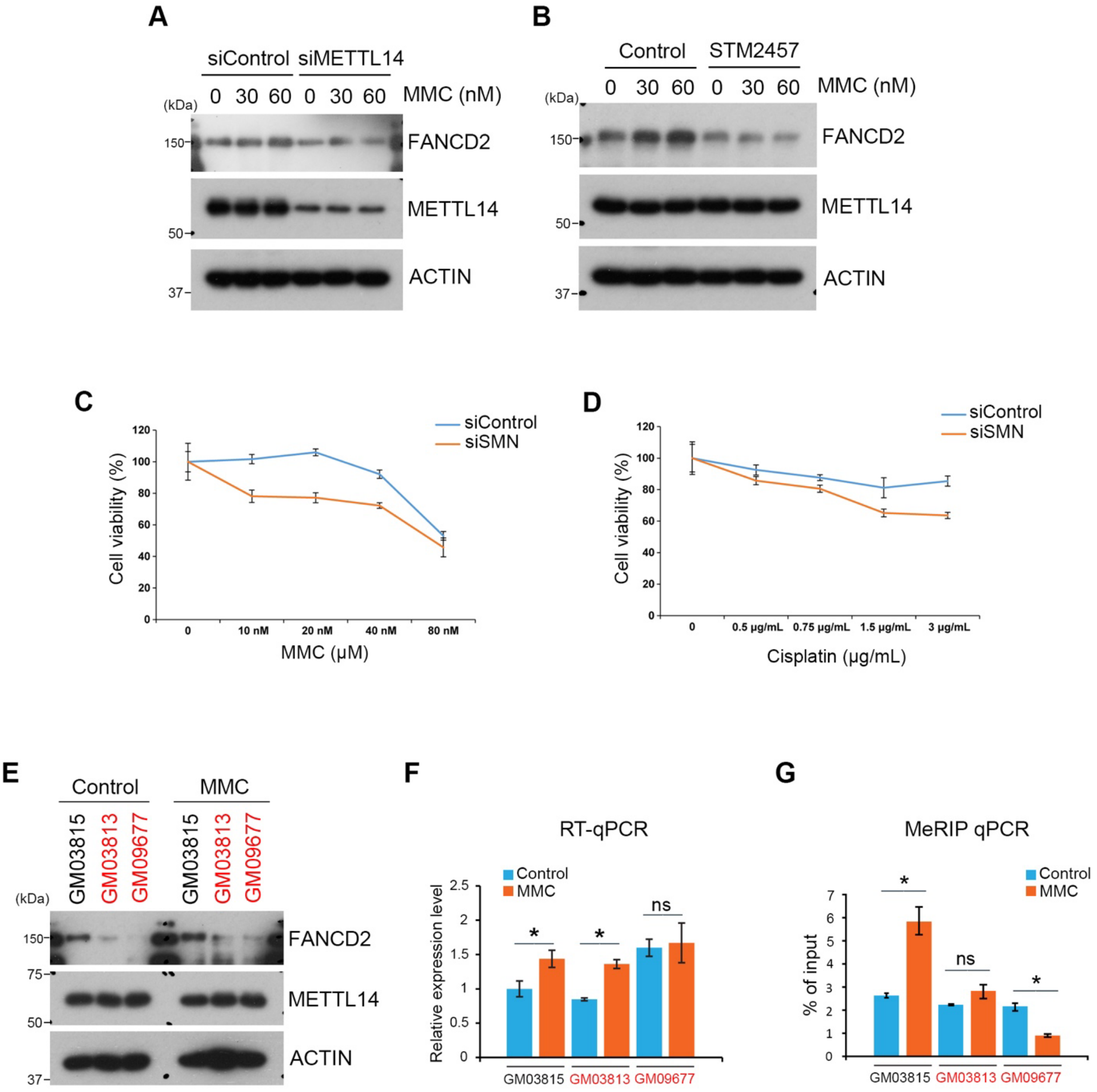

Appendix Figure S4. SMN-mediated m⁶A regulation is critical for cellular response to DNA damage.

- **(A)** Knockdown METTL14 dampens Mitomycin C (MMC)-induced FANCD2 expression. HeLa cells were transfected with control siRNA (siControl) and METTL14-specific siRNA (siMETTL14) and subjected to a dosage-dependent MMC treatment for 72 hours. The total cell lysates were subjected to western blot analysis using indicated antibodies.
- **(B)** Inhibition of m^6A deposition dampens Mitomycin C (MMC)-induced FANCD2 expression. HeLa cells were treated with either vehicle control or METTL3 inhibitor (STM2457) and subjected to a dosage-dependent MMC treatment for 72 hours. The total cell lysates were subjected to western blot analysis using indicated antibodies.
- **(C)** Knockdown of SMN sensitizes HeLa cells to MMC-induced cell death. Control (siControl) and SMN knockdown (siSMN) HeLa cells were treated with increasing dosages of MMC for 3 days. The viability of the cells was quantified using the CCK-8 assay.
- **(D)** Knockdown of SMN sensitizes HeLa cells to Cisplatin-induced cell death. Control (siControl) and SMN knockdown (siSMN) HeLa cells were treated with increasing dosages of Cisplatin for 3 days. The viability of the cells was quantified using the CCK-8 assay.
- **(E)** SMA-patient derived fibroblasts show dampened Mitomycin C (MMC)-induced FANCD2 expression. Three SMA-patient derived fibroblasts were subjected to MMC treatment (60 nM) for 72 hours. The total cell lysates were subjected to western blot analysis using indicated antibodies.
- **(F)** Detection of FANCD2 mRNA expression among the three SMA-patient derived fibroblasts following MMC treatment. The RNA expression of FANCD2 was analyzed by RT-qPCR in SMA-patient derived fibroblast treated as described in (C). Data are shown as mean ± SD from three biological replicates. *, $p < 0.05$. ns, not significant.
- **(G)** MMC treatment induces a significant increase of m^6A deposition at FANCD2 long exon in fibroblasts from non-clinically affected individual but has marginal effects in fibroblasts from clinically affected patients. The MeRIP-qPCR was performed with RNA samples from SMA-patient derived fibroblasts treated with vehicle control or MMC as described in (C). Data are shown as mean ± SD from three biological replicates. *, $p < 0.05$. ns, not significant.

Appendix Figure S5

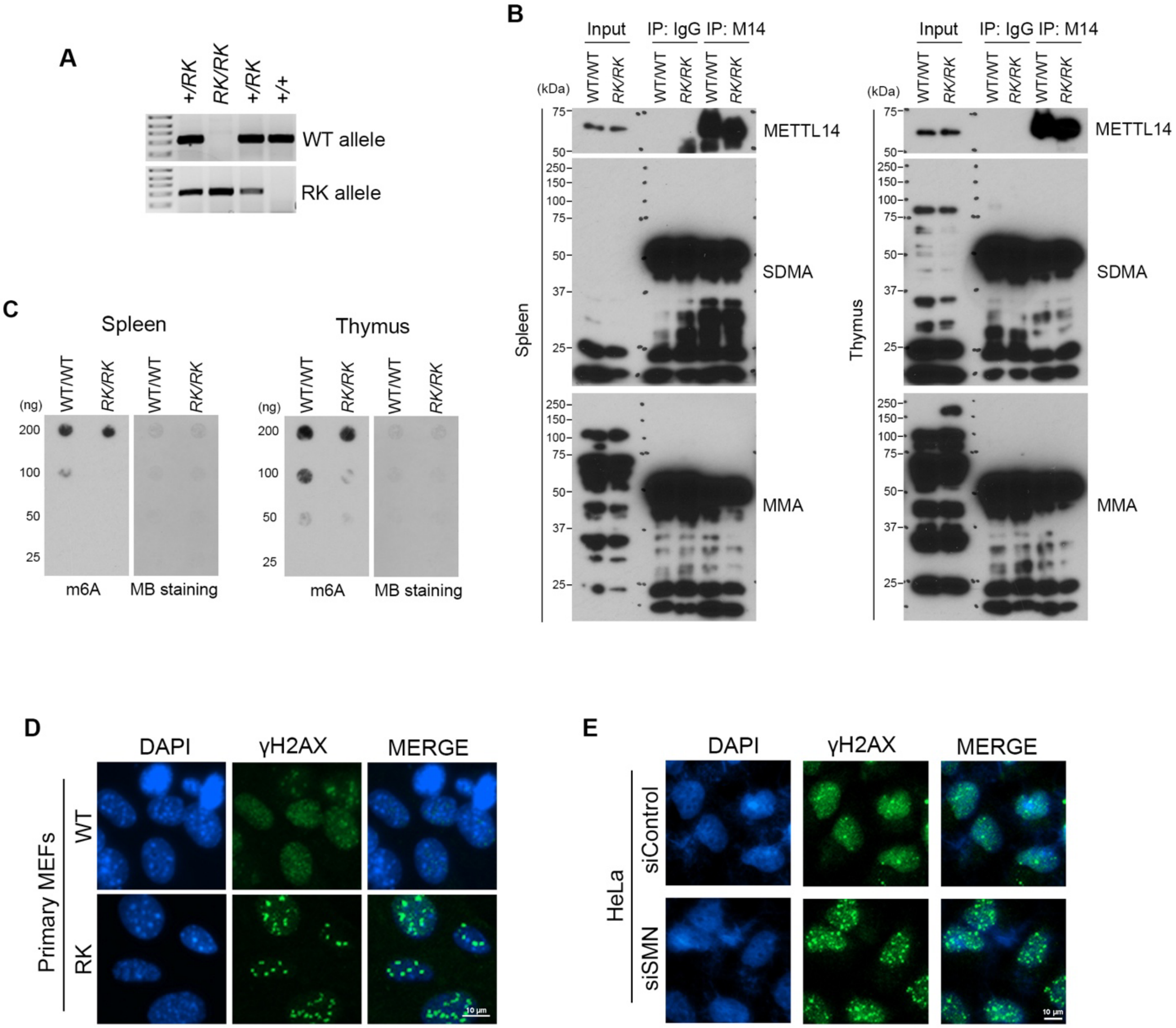

Appendix Figure S5. Tissues from Mettl14 arginine methylation deficient mouse model show reduced m^{6}A levels and increased DNA damage.

(A) Representative genotyping results of Mettl14 WT (+/+), heterozygous (+/RK), and homozygous for arginine methylation deficient (RK/RK) mutant mice.

(B) SDMA and MMA modifications of METTL14 were not detected in spleen and thymus tissues. The arginine methylation levels of Mettl14 in WT and homozygous (RK/RK) mice were detected by IP-western blot using tissue lysates from mouse spleen and thymus.

(C) Dot blot detection of m^{6}A levels of the mRNA samples purified from spleen and thymus tissues of WT and Mettl14 RK mutant (RK/RK) mice.

(D) Immunofluorescence detection of DNA damage response marked by phosphorylation of histone H2AX on serine 139 (γH2AX) in WT and Mettl14 RK mutant MEFs. DAPI staining indicates the nucleus.

(E) Immunofluorescence detection of DNA damage response marked γH2AX in HeLa cells transfected control siRNA (siControl) and SMN-specific siRNA (siSMN). DAPI staining indicates the nucleus.

Appendix Figure S6

A

Genotypes from Het x Het

	number	+/+ (25%)	+/RK (50%)	RK/RK (25%)
**P14-P21**	397	113 (28%)	243 (62%)	41 (10%)
**E12.5**	24	7 (29%)	14 (58%)	3 (13%)

	number	+/+ (25%)	+/RK (50%)	RK/RK (25%)
**Male**	208	53 (26%)	131 (63%)	24 (11%)
**Female**	189	60 (32%)	112 (59%)	17 (9%)

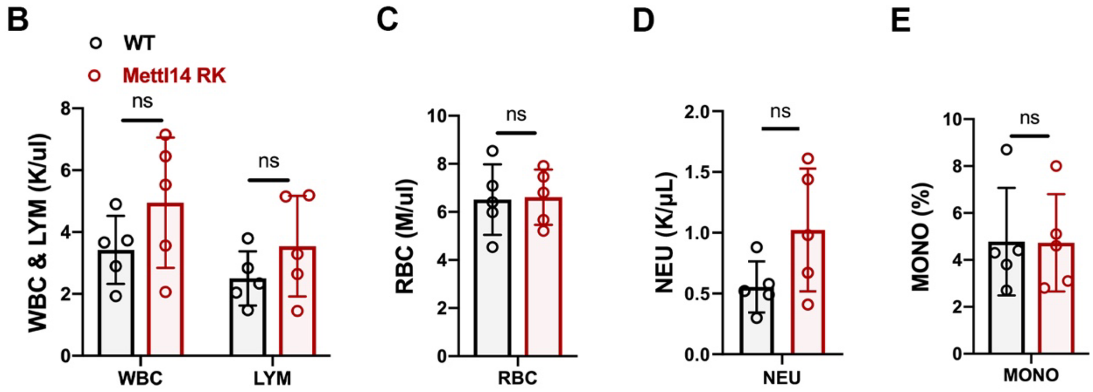

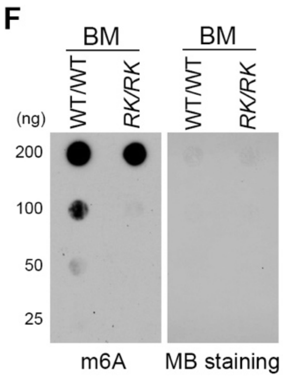

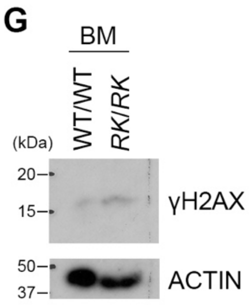

Appendix Figure S6. Characterization of Mettl14 RK mutant mice.

- **(A)** Mettl14 arginine methylation deficiency causes partial embryonic lethality. The numbers of Mettl14 WT (+/+), heterozygous (+/RK), and homozygous for arginine methylation deficient (RK/RK) mutant mice are presented for the indicated stages. P14-P21 refers to postnatal day 14 – 21. E12.5 refers to embryonic day 12.5.
- **(B)** – **(E)** Complete blood counts of peripheral blood from WT and Mettl14 RK mice on white blood cell (WBC), lymphocyte (LYM), red blood cell (RBC), neutrophil (NEU), and monocyte (MONO). No significant changes were observed (n=5 for each genotype).
- **(F)** Dot blot detection of m^6A levels of the mRNA samples purified from bone marrow tissues of WT and Mettl14 RK mutant (RK/RK) mice.
- **(G)** Western blot detection of phosphorylation of histone H2AX on serine 139 (γH2AX) in total cell lysates of bone marrow tissues from WT and Mettl14 RK mutant (RK/RK) mice.

Appendix Figure S7

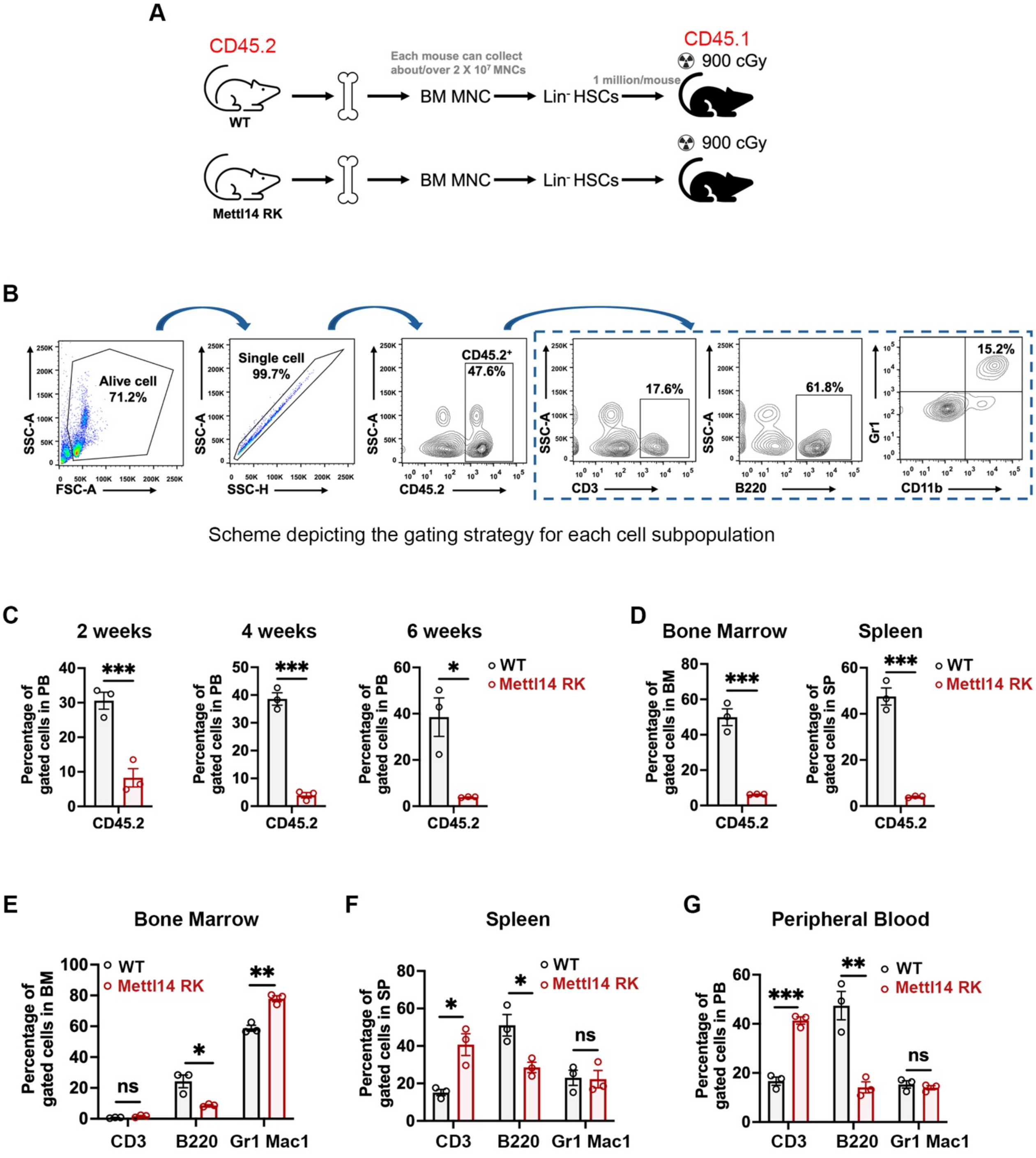

Appendix Figure S7. Impacts of Mettl14 arginine methylation deficiency on hematopoiesis revealed by hematopoietic stem cell reconstitution.

(A) Schematic diagram illustrating the hematopoietic stem cell reconstitution assays in lethally irradiated $CD45.1^+$ recipient mice with CD45.2+ WT and Mettl14 RK Lin- HSCs.

(B) Gating strategy used to identify each cell subpopulation.

(C) Effect of Mettl14 RK on the $CD45.2^+$ population in peripheral blood following HSC reconstitution, as determined by flow cytometry. Samples were collected at 2-, 4-, and 6-weeks post-transplantation.

(D) Effect of Mettl14 RK on the $CD45.2^+$ population in bone marrow and spleen following HSC reconstitution, as determined by flow cytometry. Samples were collected at 6 weeks post-transplantation.

(E) Effect of Mettl14 RK on the frequencies of T-lymphoid ($CD3^+$), B-lymphoid ($B220^+$), and myeloid ($Gr1^+Mac1^+$) cells in CD45.2+ bone marrow mononuclear cells following HSC reconstitution, as determined by flow cytometry. Samples were collected at 6 weeks post-transplantation.

(F) Effect of Mettl14 RK on the frequencies of T-lymphoid ($CD3^+$), B-lymphoid ($B220^+$), and myeloid ($Gr1^+Mac1^+$) cells in CD45.2+ spleen mononuclear cells following HSC reconstitution, as determined by flow cytometry. Samples were collected at 6 weeks post-transplantation.

(G) Effect of Mettl14 RK on the frequencies of T-lymphoid ($CD3^+$), B-lymphoid ($B220^+$), and myeloid ($Gr1^+Mac1^+$) cells in CD45.2+ peripheral blood mononuclear cells following HSC reconstitution, as determined by flow cytometry. Samples were collected at 6 weeks post-transplantation. Data in C-G) are shown as mean ± SEM. Unpaired Student's t-test (C-G). ns, not significant; *P < 0.05; ** P < 0.01; *** P < 0.001.

Appendix Table S1. Primers used in this study

Primer Name	Primer sequence (5’-3’)
**RT-qPCR primers**	
YTHDF1 Forward	ACCTGTCCAGCTATTACCCG
YTHDF1 Reverse	TGGTGAGGTATGGAATCGGAG
YTHDF2 Forward	AGCCCCACTTCCTACCAGATG
YTHDF2 Reverse	TGAGAACTGTTATTTCCCCATGC
YTHDF3 Forward	TCAGAGTAACAGCTATCCACCA
YTHDF3 Reverse	GGTTGTCAGATATGGCATAGGCT
YTHDC1 Forward	GAGGGCCAAATCTCCTACGC
YTHDC1 Reverse	GTCTCATGGTCAGAGCCATATTC
YTHDC2 Forward	CAAAACATGCTGTTAGGAGCCT
YTHDC2 Reverse	CCACTTGTCTTGCTCATTTCCC
WTAP Forward	CTTCCCAAGAAGGTTCGATTGA
WTAP Reverse	TCAGACTCTCTTAGGCCAGTTAC
METTL3 Forward	TTGTCTCCAACCTTCCGTAGT
METTL3 Reverse	CCAGATCAGAGAGGTGGTGTAG
METTL14 Forward	AGTGCCGACAGCATTGGTG
METTL14 Reverse	GGAGCAGAGGTATCATAGGAAGC
FTO Forward	ACTTGGCTCCCTTATCTGACC
FTO Reverse	TGTGCAGTGTGAGAAAGGCTT
ALKBH5 Forward	ATGCACCCCGGTTGGAAAC
ALKBH5 Reverse	GACTTGCGCCAGTAGTTCTCA
FANCD2 Forward	AAAACGGGAGAGAGTCAGAATCA
FANCD2 Reverse	ACGCTCACAAGACAAAAGGCA
FANCM Forward	TTCAAGGTTTGTAACGGGAATGC
FANCM Reverse	AAGTGACTCTGTCTCTTTGGGAC
ATRIP Forward	AGAGGTACTTCAGGCACAATACA
ATRIP Reverse	TGCAATGATTGGAGCTTTTTGGA
PALB2 Forward	AGGATCTCTCACCGCAGCTAA
PALB2 Reverse	TCAGGCCCAACATCAAGTGTG
BLM Forward	ACCGAAAAAGCCTAAAAAGGTGG
BLM Reverse	ATGGGTGGTGCTTTCTGATCC
BRCA1 Forward	TAGAACAGCATGGGAGCCAG
BRCA1 Reverse	CTGGATTTCGCAGGTCCTCA
BRCA2 Forward	ATCAGCTGGCTTCAACTCCA
BRCA2 Reverse	ACAGGAGATTGGTACAGCGG
SMN Forward	CCTGTGTTGTGGTTTACACTGG
SMN Reverse	GGGGGAATTATCTTTCCTGGTCC
ACTIN Forward	AGACCTGTACGCCAACACAG
ACTIN Reverse	GGAGCAATGATCTTGATCTTCA
**MeRIP-qPCR primers**	
FANCD2 Forward	AGCACTGTACGGACTGGAAG
FANCD2 Reverse	GTCCTGAGAAAACAGCAGCG
PALB2 Forward	AAGGCCTTCTGTTTCCTGCA
PALB2 Reverse	CCTCCACGGCTACTTTCCTC
ATRIP Forward	ACACATTCCTCCTGCGTGAG

ATRIP Reverse	AGAAGCCTTCCAGGATGCAC
FANCM Forward	TATTCTCCTCCGCCTCTCAGT
FANCM Reverse	TGCTCTGCACAGGGTAAGAAA
BLM Forward	CAACTGGGCTGAAACACCAAG
BLM Reverse	CACAGCAGTGCTTGTGAGAAC
BRCA1 Forward	CCGAAGAGGGGCCAAGAAAT
BRCA1 Reverse	GTTGGAAGCAGGGAAGCTCT
BRCA2 Forward	CAGAAGCCCTTTGAGAGTGGA
BRCA2 Reverse	TGAGACCATTCACAGGCCAAA
**Mettl14WT/RK mouse genotyping primers**	
Mettl14WT Forward	CCCGCTTTATTTCAGGCTGGCTC
Mettl14WT Reverse	GCCTCTGTGCGTGCCTCCACGG
Mettl14RK Forward	CCCGCTTTATTTCAGGCTGGCTC
Mettl14RK Reverse	GCCTTTGTGCGTGCCTCCCTTG
**Splicing primers**	
BLM P1-Forward	TGTGGGAACGAACTGCTTCA
BLM P1-Reverse	CCTGGTGGCAGAGAATCCTG
BLM P2-Forward	GCAGCCAGCAAATCTTCCAC
BLM P2-Reverse	AGTCTTCACCAAGCAGTGCA
BLM P3-Forward	TGTGGGAACGAACTGCTTCA
BLM P3-Reverse	AGTCTTCACCAAGCAGTGCA
ATRIP P1-Forward	ATCCCTAAGCCTTTGCCACC
ATRIP P1-Reverse	AGTGCAGTCACAGAGAAGCC
ATRIP P2-Forward	GGCTTCTCTGTGACTGCACT
ATRIP P2-Reverse	GCAGCACTTGGAACACACAC
ATRIP P3-Forward	ATCCCTAAGCCTTTGCCACC
ATRIP P3-Reverse	GCAGCACTTGGAACACACAC
FANCM P1-Forward	TCAGATCGATGCCGCCATTT
FANCM P1-Reverse	TCACTGAGGATGAACTGCCTTG
FANCM P2-Forward	GCCACTGAGTGCAGCAAAAA
FANCM P2-Reverse	TCGGAAAATCTCGTCATCTTCACT
FANCM P3-Forward	TCAGATCGATGCCGCCATTT
FANCM P3-Reverse	TCGGAAAATCTCGTCATCTTCACT
SMN-Forward	CATGGTACATGAGTGGCTATCATACTG
SMN-Reverse	TGGTGTCATTTAGTGCTGCTCTATG
ACTIN-Forward	TCCTTCTGCATCCTGTCGGC
ACTIN-Reverse	AAGAGATGGCCACGGCTGCT
