# Supplementary figures and images for: Arginine methylation-dependent METTL14-SMN interaction regulates RNA m6A homeostasis (part 1 of 2)
Source: EMBO Rep. 2025 Oct 6;26(22):5483–500. doi: 10.1038/s44319-025-00590-7 (PMC12635257; doi:10.1038/s44319-025-00590-7)

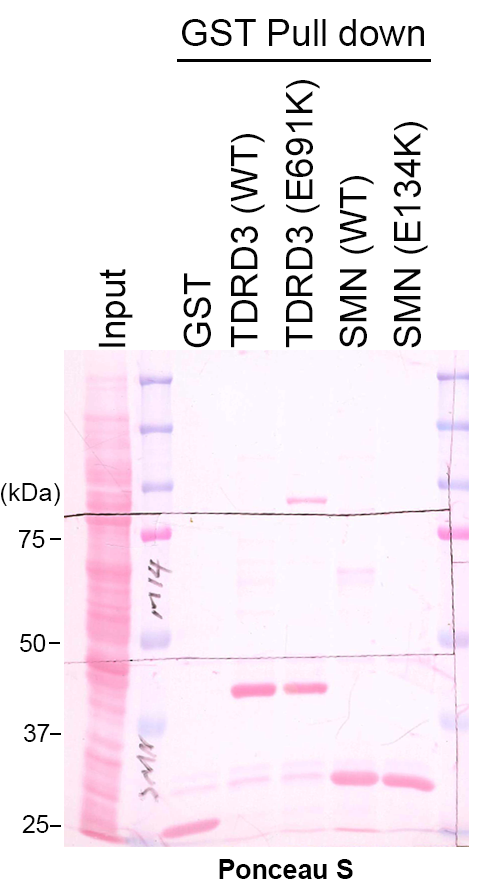

Supplement: Supplementary file 3 — Source data Fig. 1 [file 44319_2025_590_MOESM3_ESM.zip › Figure 1/1A/Ponceau S.tif]

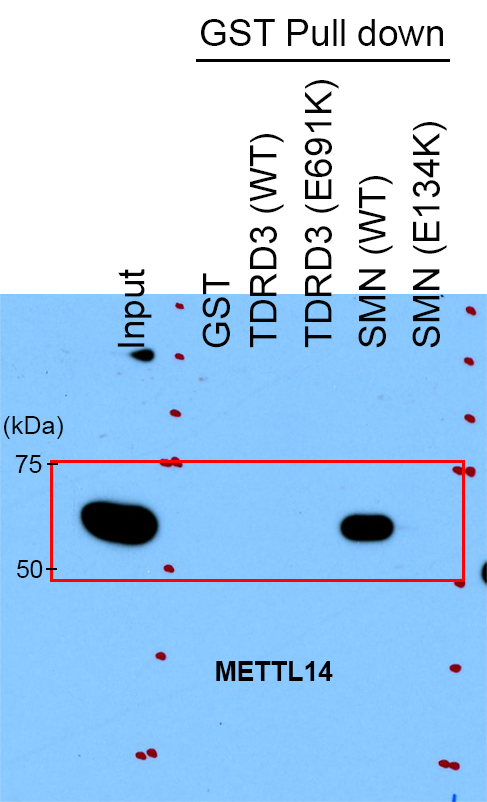

Supplement: Supplementary file 3 — Source data Fig. 1 [file 44319_2025_590_MOESM3_ESM.zip › Figure 1/1A/Western METTL14.tif]

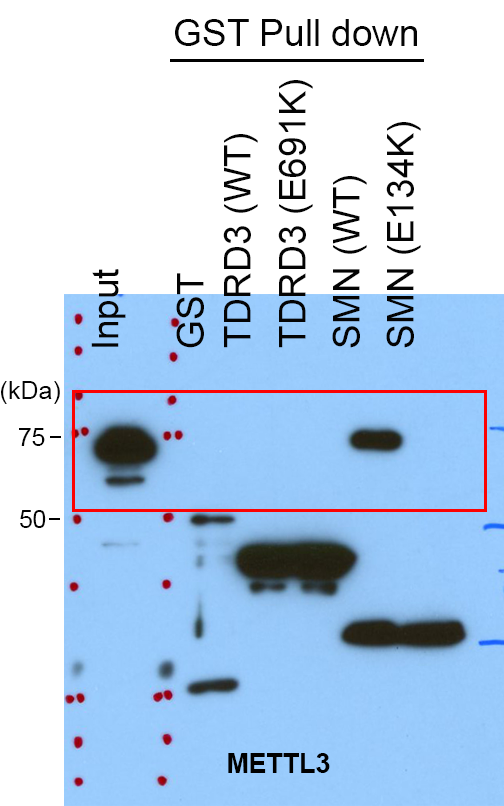

Supplement: Supplementary file 3 — Source data Fig. 1 [file 44319_2025_590_MOESM3_ESM.zip › Figure 1/1A/Western METTL3.tif]

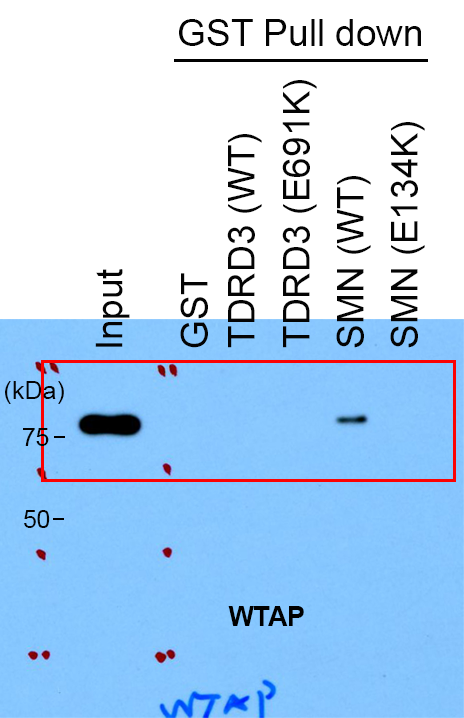

Supplement: Supplementary file 3 — Source data Fig. 1 [file 44319_2025_590_MOESM3_ESM.zip › Figure 1/1A/Western WTAP.tif]

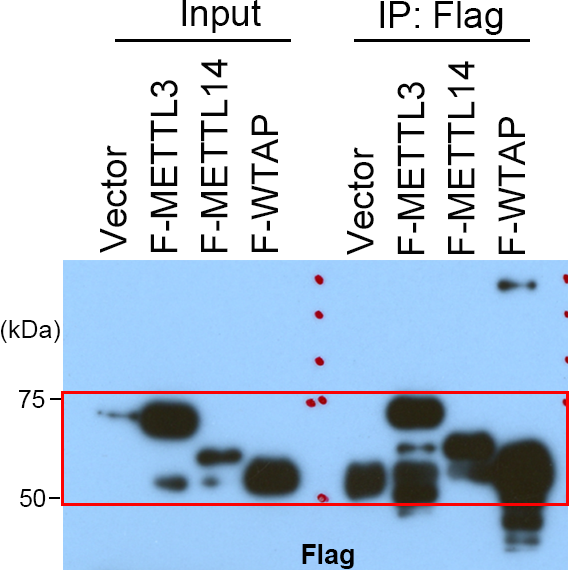

Supplement: Supplementary file 3 — Source data Fig. 1 [file 44319_2025_590_MOESM3_ESM.zip › Figure 1/1B/Western Flag.tif]

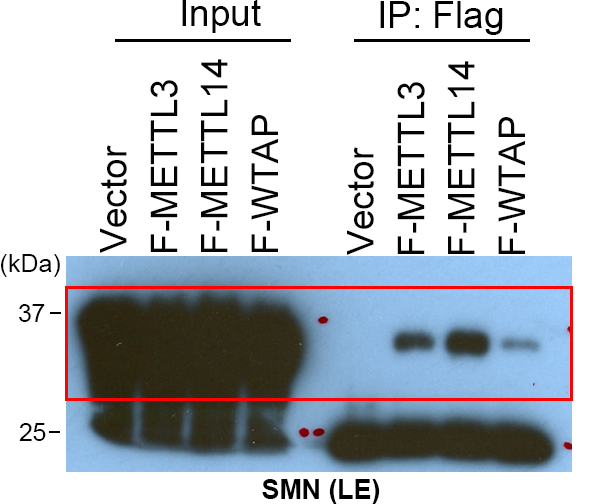

Supplement: Supplementary file 3 — Source data Fig. 1 [file 44319_2025_590_MOESM3_ESM.zip › Figure 1/1B/Western SMN (LE).tif]

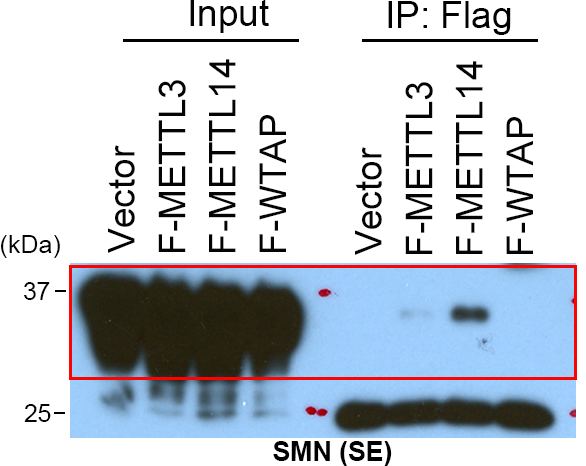

Supplement: Supplementary file 3 — Source data Fig. 1 [file 44319_2025_590_MOESM3_ESM.zip › Figure 1/1B/Western SMN (SE).tif]

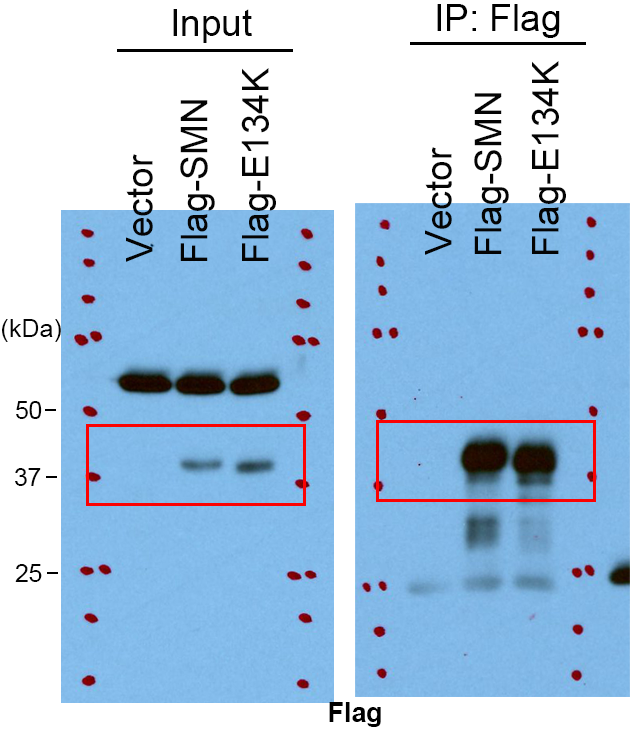

Supplement: Supplementary file 3 — Source data Fig. 1 [file 44319_2025_590_MOESM3_ESM.zip › Figure 1/1E/Western Flag.tif]

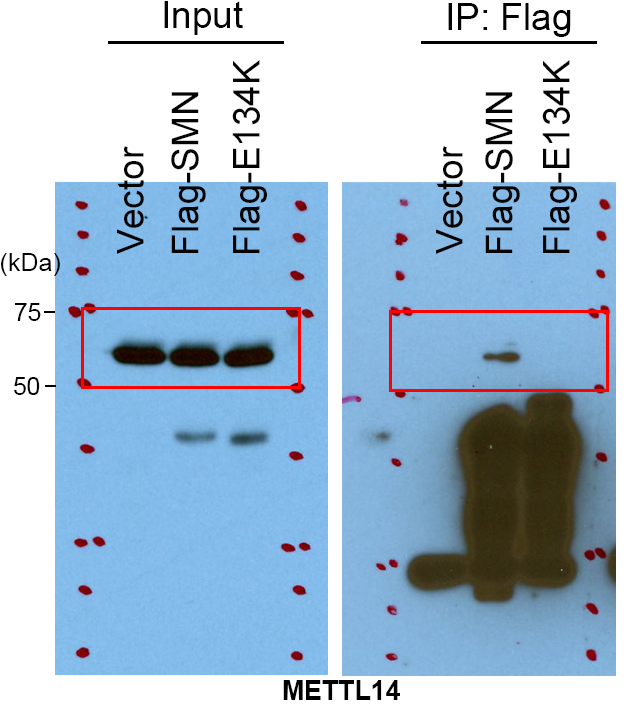

Supplement: Supplementary file 3 — Source data Fig. 1 [file 44319_2025_590_MOESM3_ESM.zip › Figure 1/1E/Western METTL14.tif]

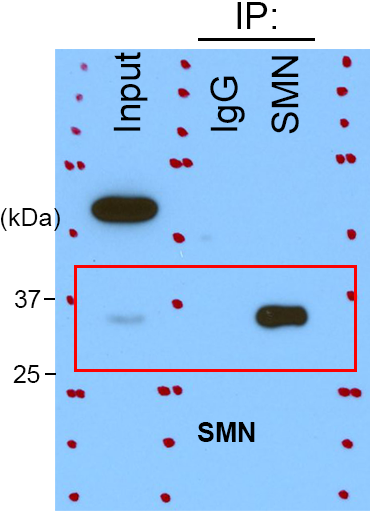

Supplement: Supplementary file 3 — Source data Fig. 1 [file 44319_2025_590_MOESM3_ESM.zip › Figure 1/1D/Western SMN.tif]

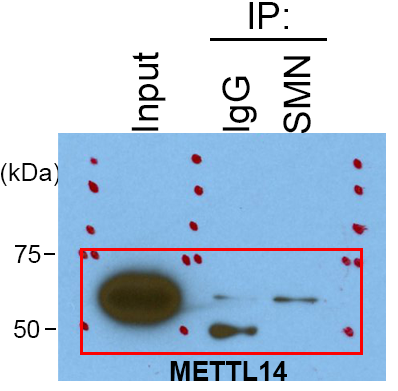

Supplement: Supplementary file 3 — Source data Fig. 1 [file 44319_2025_590_MOESM3_ESM.zip › Figure 1/1D/Western METTL14.tif]

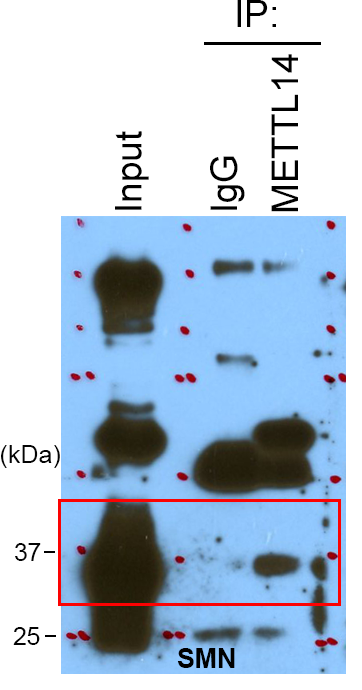

Supplement: Supplementary file 3 — Source data Fig. 1 [file 44319_2025_590_MOESM3_ESM.zip › Figure 1/1C/Western SMN.tif]

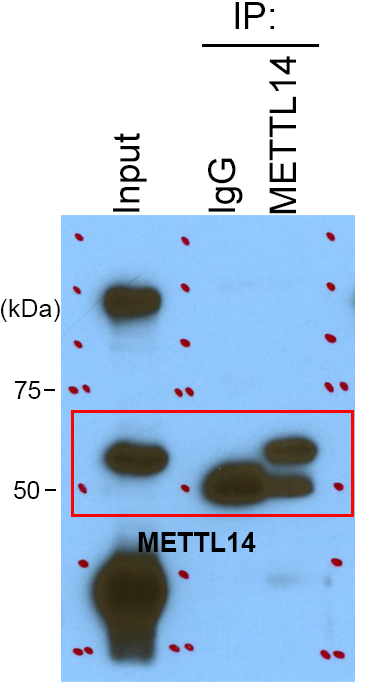

Supplement: Supplementary file 3 — Source data Fig. 1 [file 44319_2025_590_MOESM3_ESM.zip › Figure 1/1C/Western METTL14.tif]

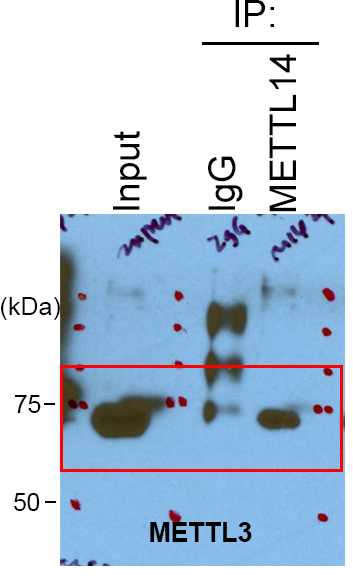

Supplement: Supplementary file 3 — Source data Fig. 1 [file 44319_2025_590_MOESM3_ESM.zip › Figure 1/1C/Western METTL3.tif]

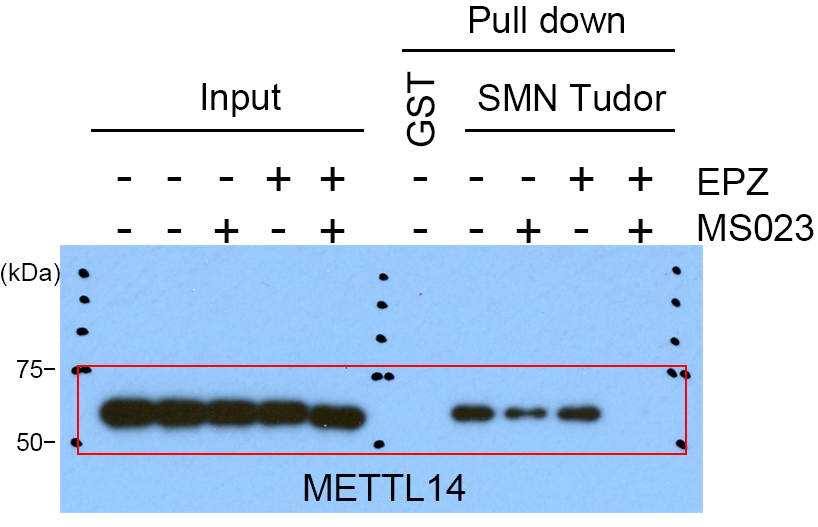

Supplement: Supplementary file 4 — Source data Fig. 2 [file 44319_2025_590_MOESM4_ESM.zip › Figure 2/2G/Western METTL14.tif]

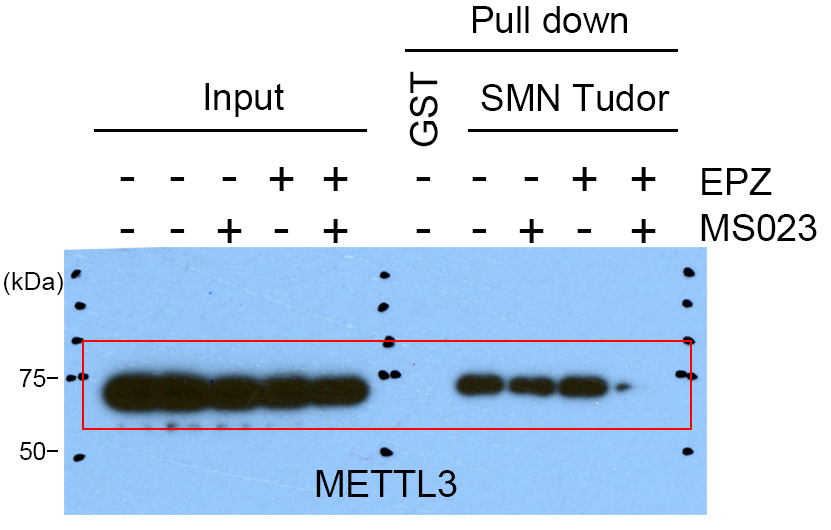

Supplement: Supplementary file 4 — Source data Fig. 2 [file 44319_2025_590_MOESM4_ESM.zip › Figure 2/2G/Western METTL3.tif]

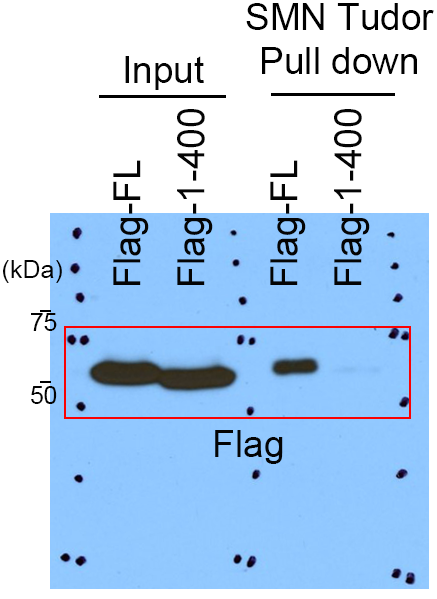

Supplement: Supplementary file 4 — Source data Fig. 2 [file 44319_2025_590_MOESM4_ESM.zip › Figure 2/2A/Western Flag.tif]

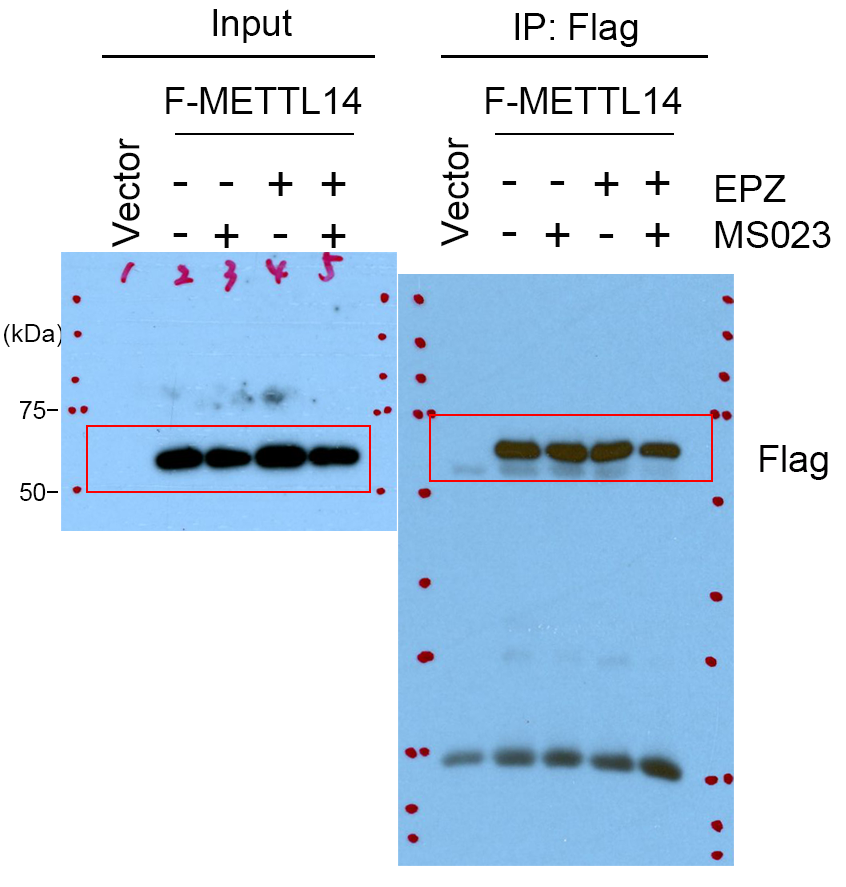

Supplement: Supplementary file 4 — Source data Fig. 2 [file 44319_2025_590_MOESM4_ESM.zip › Figure 2/2F/Western Flag.tif]

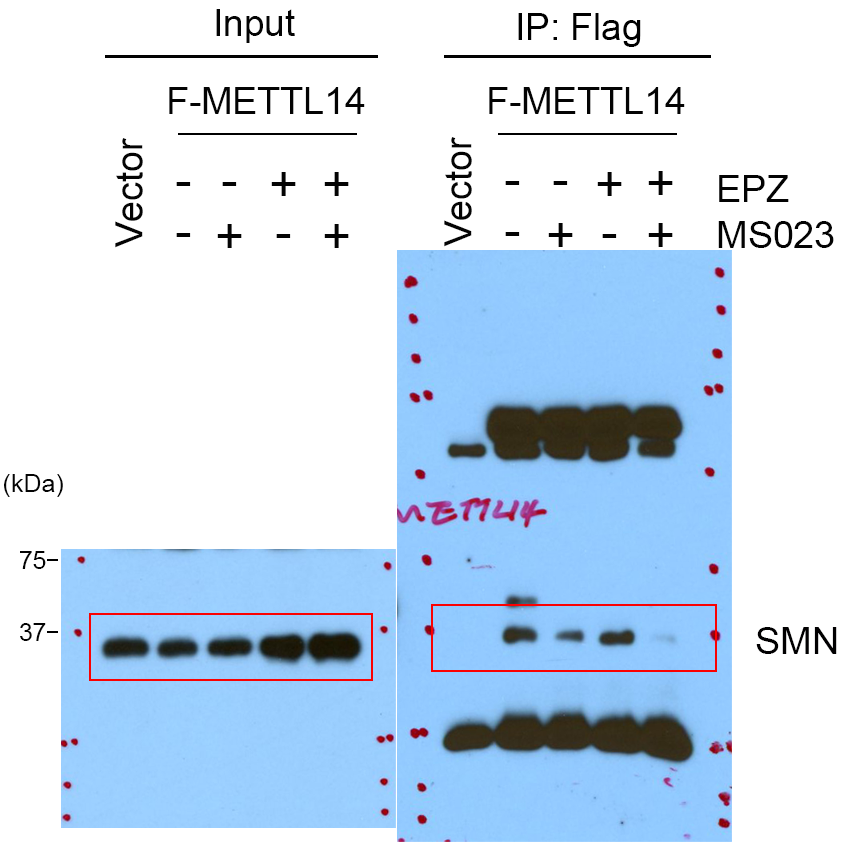

Supplement: Supplementary file 4 — Source data Fig. 2 [file 44319_2025_590_MOESM4_ESM.zip › Figure 2/2F/Western SMN.tif]

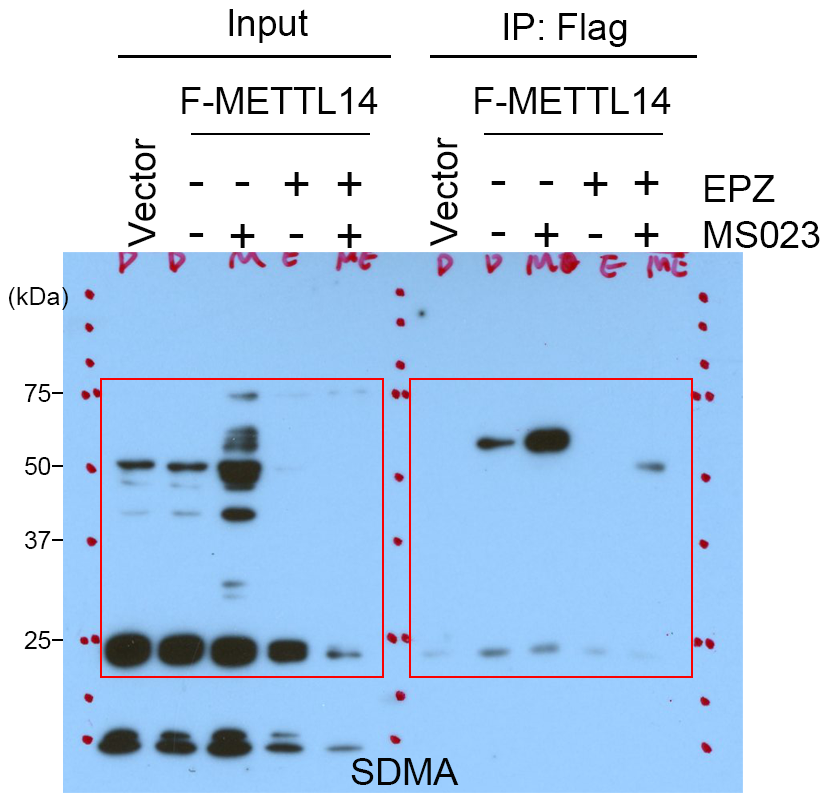

Supplement: Supplementary file 4 — Source data Fig. 2 [file 44319_2025_590_MOESM4_ESM.zip › Figure 2/2F/Western SDMA.tif]

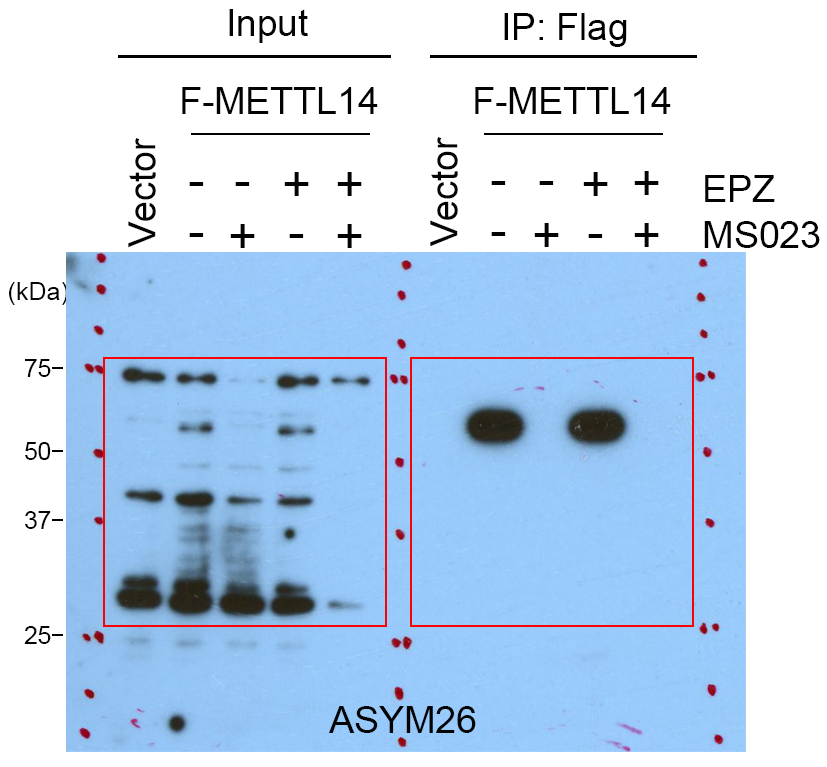

Supplement: Supplementary file 4 — Source data Fig. 2 [file 44319_2025_590_MOESM4_ESM.zip › Figure 2/2F/Western ASYM26.tif]

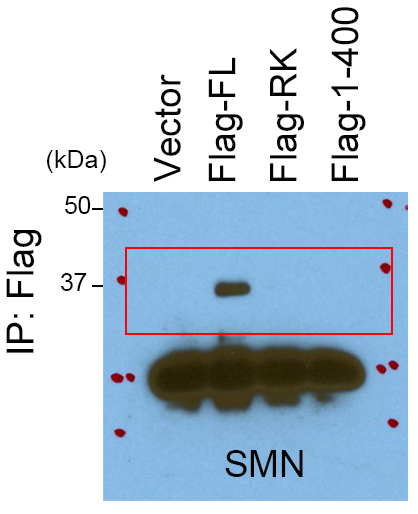

Supplement: Supplementary file 4 — Source data Fig. 2 [file 44319_2025_590_MOESM4_ESM.zip › Figure 2/2C/Western SMN (IP).tif]

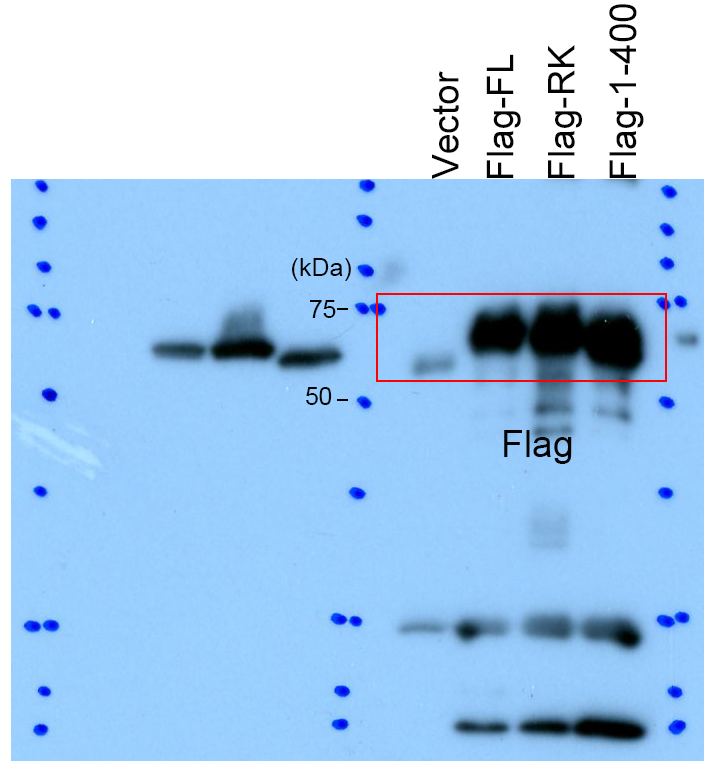

Supplement: Supplementary file 4 — Source data Fig. 2 [file 44319_2025_590_MOESM4_ESM.zip › Figure 2/2C/Western Flag (IP).tif]

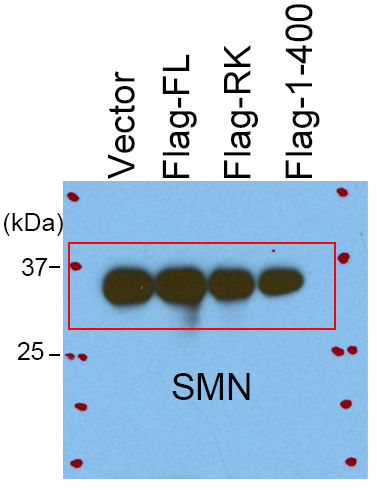

Supplement: Supplementary file 4 — Source data Fig. 2 [file 44319_2025_590_MOESM4_ESM.zip › Figure 2/2C/Western SMN (Input).tif]

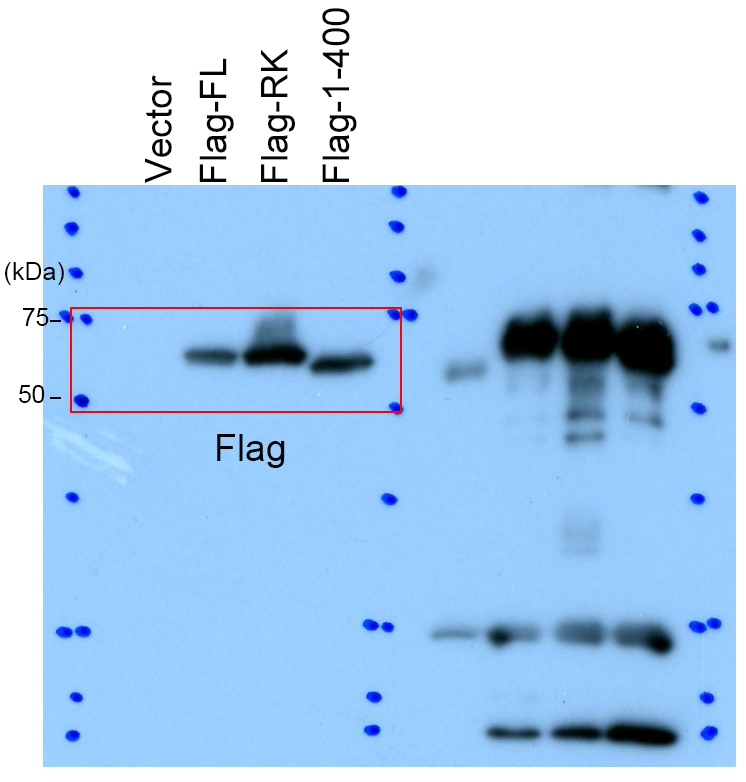

Supplement: Supplementary file 4 — Source data Fig. 2 [file 44319_2025_590_MOESM4_ESM.zip › Figure 2/2C/Western Flag (Input).tif]

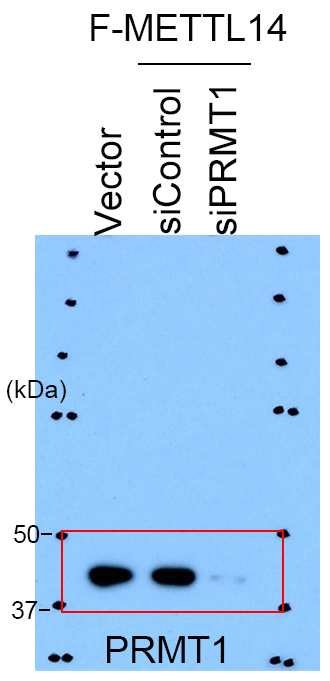

Supplement: Supplementary file 4 — Source data Fig. 2 [file 44319_2025_590_MOESM4_ESM.zip › Figure 2/2D/Western PRMT1.tif]

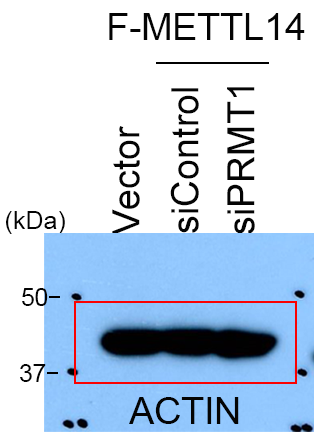

Supplement: Supplementary file 4 — Source data Fig. 2 [file 44319_2025_590_MOESM4_ESM.zip › Figure 2/2D/Western ACTIN.tif]

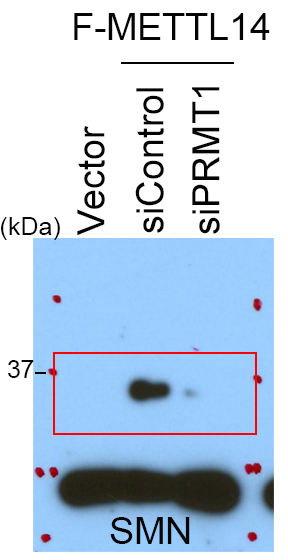

Supplement: Supplementary file 4 — Source data Fig. 2 [file 44319_2025_590_MOESM4_ESM.zip › Figure 2/2D/Western SMN (IP).tif]

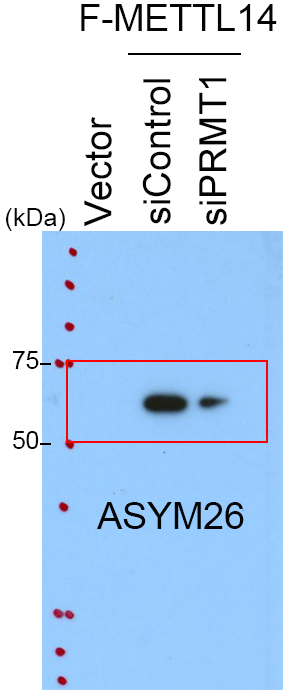

Supplement: Supplementary file 4 — Source data Fig. 2 [file 44319_2025_590_MOESM4_ESM.zip › Figure 2/2D/Western ASYM26.tif]

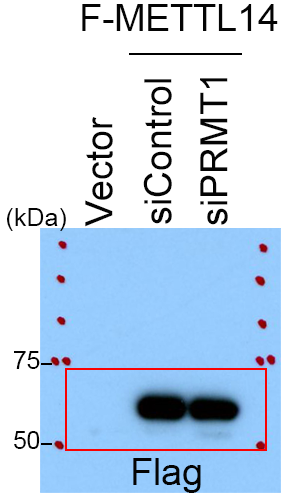

Supplement: Supplementary file 4 — Source data Fig. 2 [file 44319_2025_590_MOESM4_ESM.zip › Figure 2/2D/Western Flag (IP).tif]

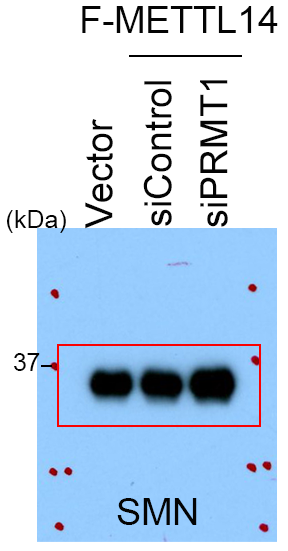

Supplement: Supplementary file 4 — Source data Fig. 2 [file 44319_2025_590_MOESM4_ESM.zip › Figure 2/2D/Western SMN (Input).tif]

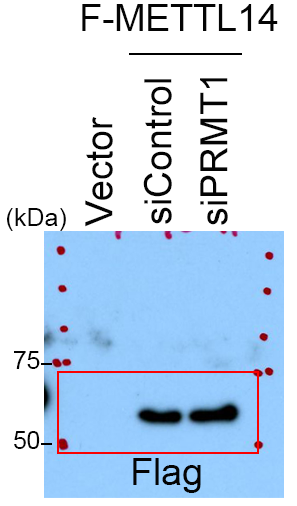

Supplement: Supplementary file 4 — Source data Fig. 2 [file 44319_2025_590_MOESM4_ESM.zip › Figure 2/2D/Western Flag (Input).tif]

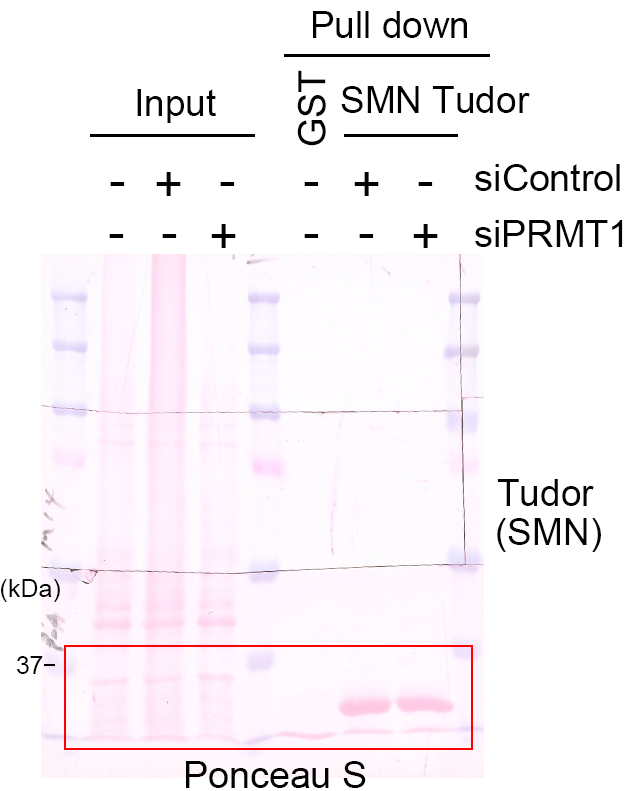

Supplement: Supplementary file 4 — Source data Fig. 2 [file 44319_2025_590_MOESM4_ESM.zip › Figure 2/2E/Ponceau S SMN Tudor.tif]

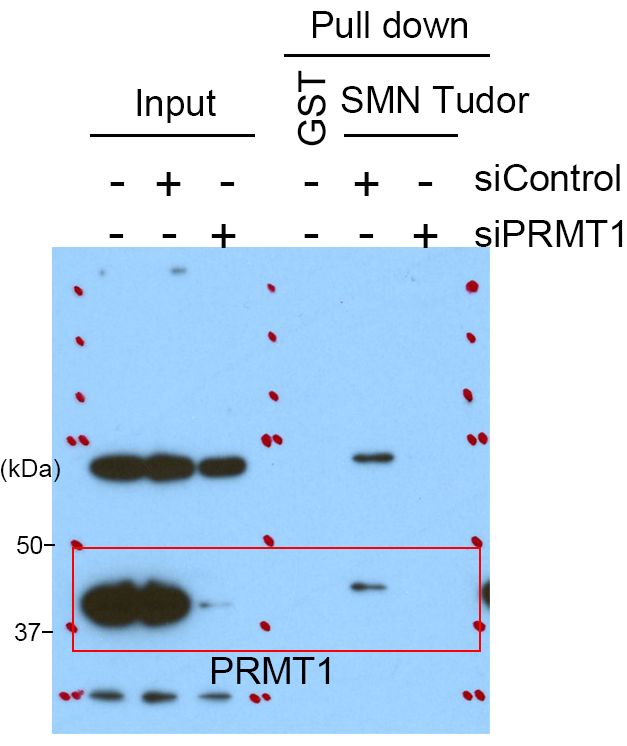

Supplement: Supplementary file 4 — Source data Fig. 2 [file 44319_2025_590_MOESM4_ESM.zip › Figure 2/2E/Western PRMT1.tif]

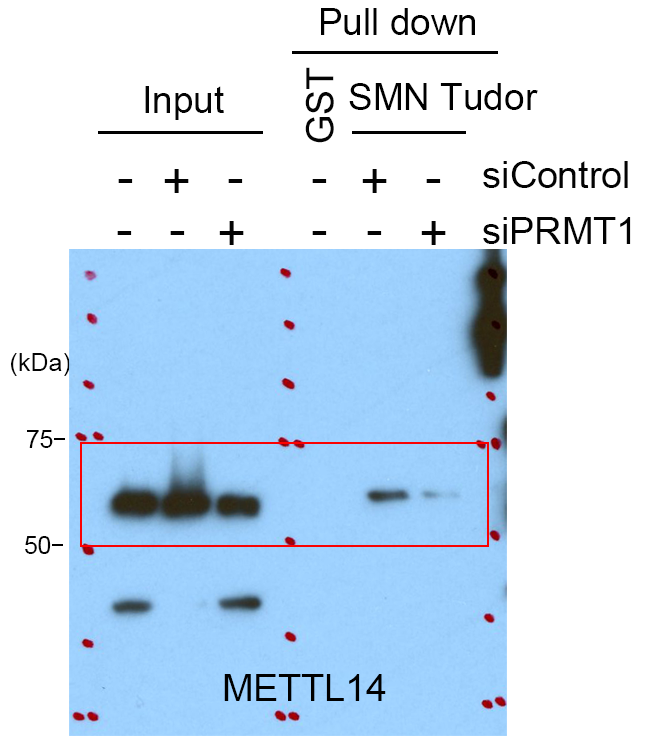

Supplement: Supplementary file 4 — Source data Fig. 2 [file 44319_2025_590_MOESM4_ESM.zip › Figure 2/2E/Western METTL14.tif]

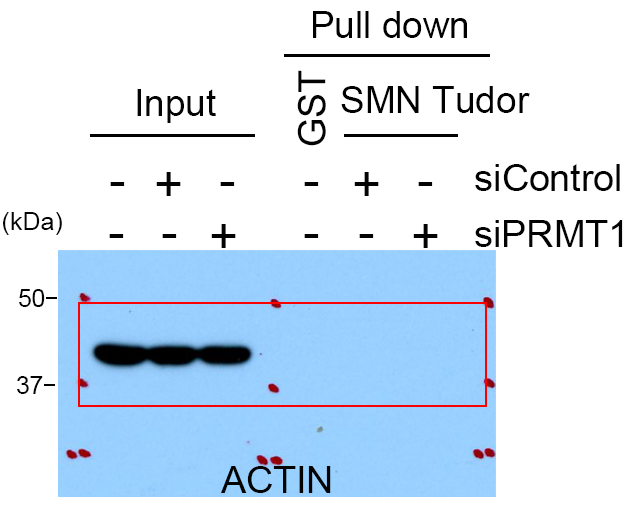

Supplement: Supplementary file 4 — Source data Fig. 2 [file 44319_2025_590_MOESM4_ESM.zip › Figure 2/2E/Western ACTIN.tif]

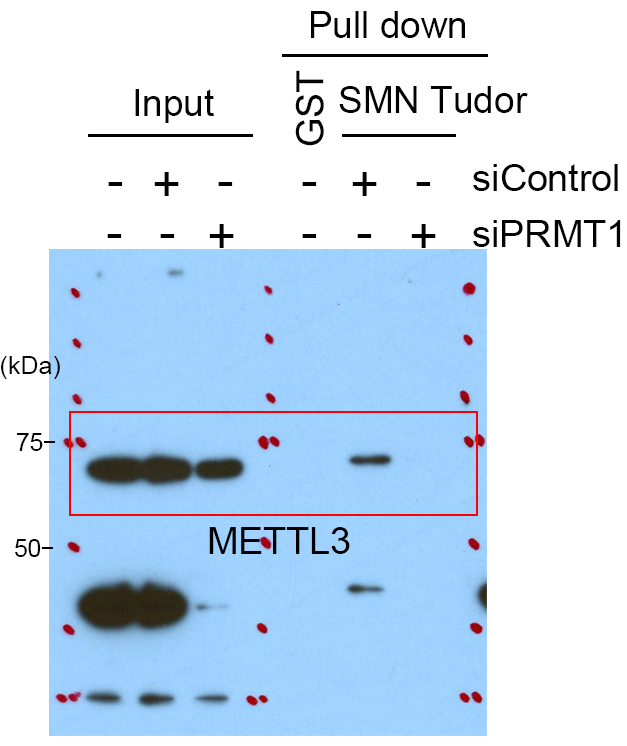

Supplement: Supplementary file 4 — Source data Fig. 2 [file 44319_2025_590_MOESM4_ESM.zip › Figure 2/2E/Western METTL3.tif]

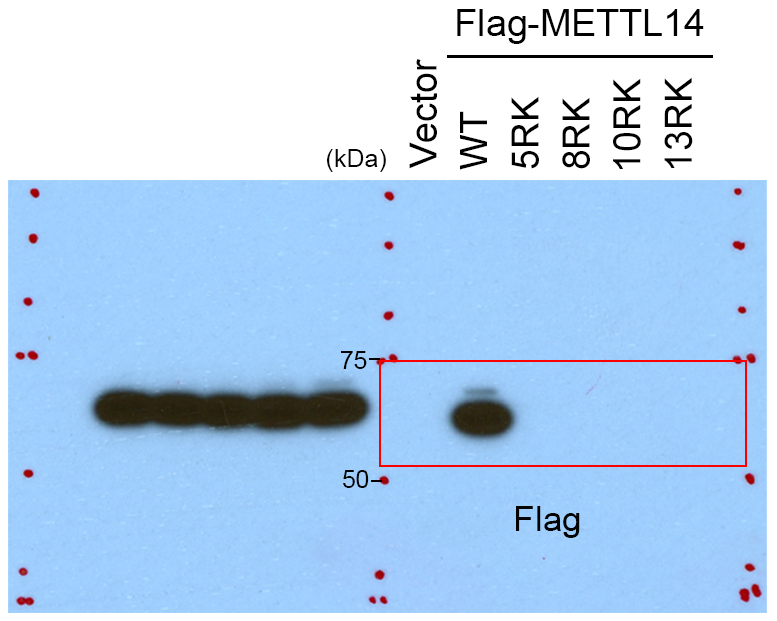

Supplement: Supplementary file 4 — Source data Fig. 2 [file 44319_2025_590_MOESM4_ESM.zip › Figure 2/2B/Western Flag (Pull down).tif]

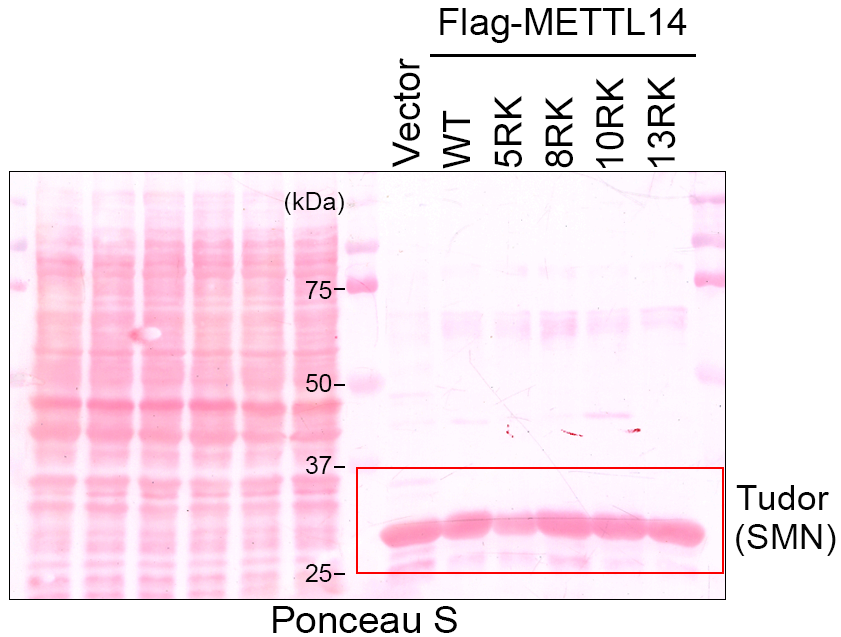

Supplement: Supplementary file 4 — Source data Fig. 2 [file 44319_2025_590_MOESM4_ESM.zip › Figure 2/2B/Ponceau S Tudor (SMN).tif]

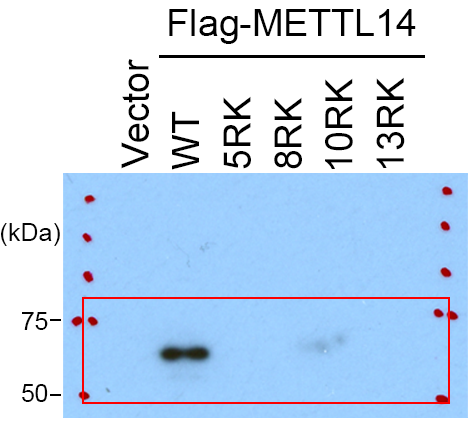

Supplement: Supplementary file 4 — Source data Fig. 2 [file 44319_2025_590_MOESM4_ESM.zip › Figure 2/2B/Western ADMA.tif]

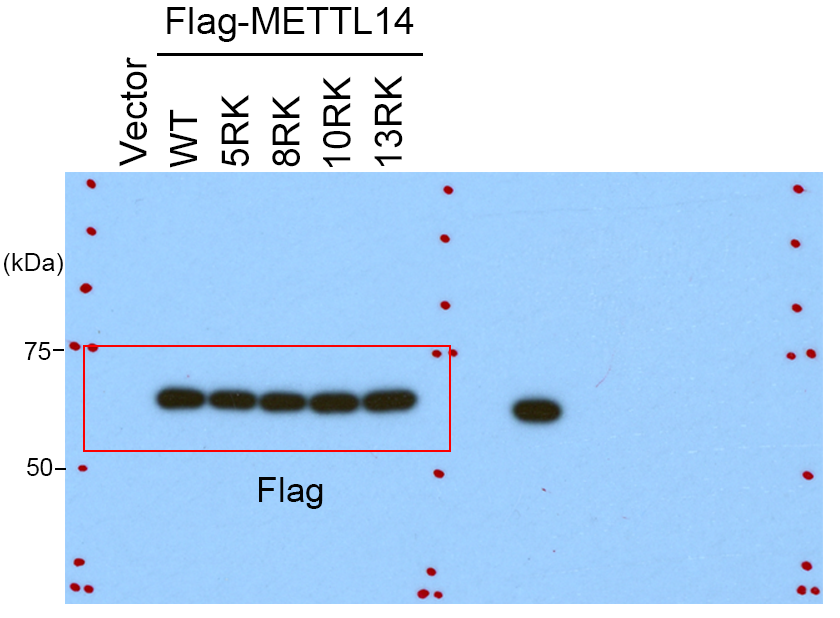

Supplement: Supplementary file 4 — Source data Fig. 2 [file 44319_2025_590_MOESM4_ESM.zip › Figure 2/2B/Western Flag (Input).tif]

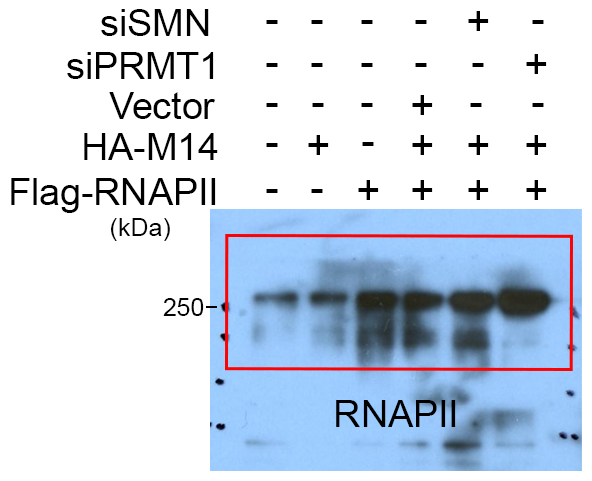

Supplement: Supplementary file 5 — Source data Fig. 3 [file 44319_2025_590_MOESM5_ESM.zip › Figure 3/3B/Western RNAP II (Input).tif]

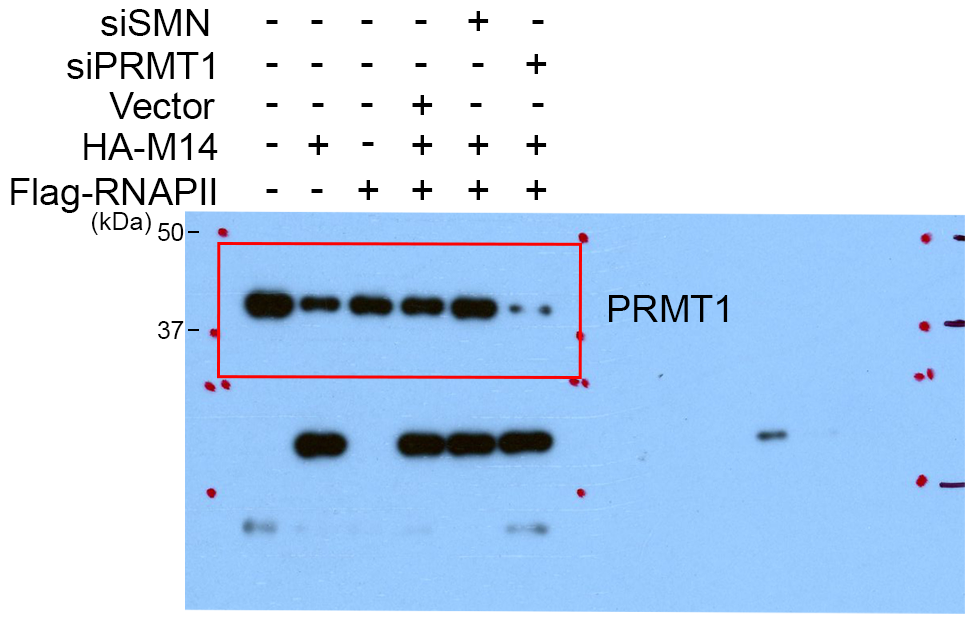

Supplement: Supplementary file 5 — Source data Fig. 3 [file 44319_2025_590_MOESM5_ESM.zip › Figure 3/3B/Western PRMT1.tif]

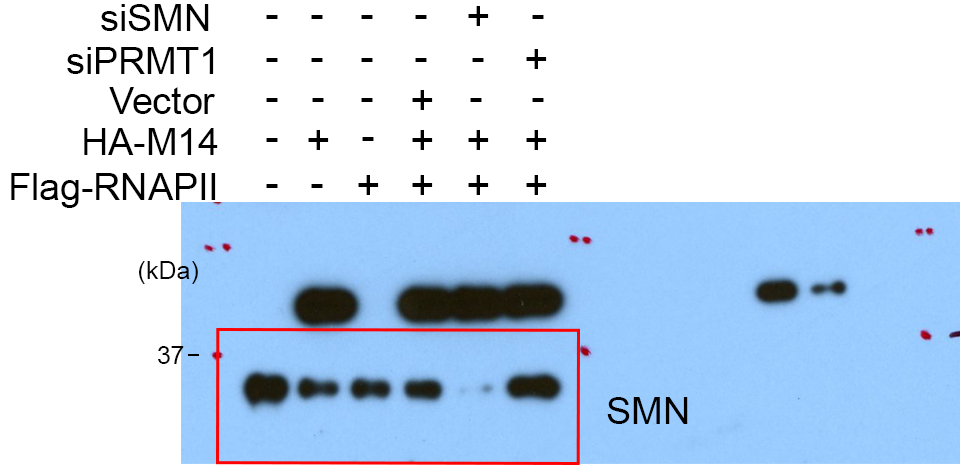

Supplement: Supplementary file 5 — Source data Fig. 3 [file 44319_2025_590_MOESM5_ESM.zip › Figure 3/3B/Western SMN.tif]

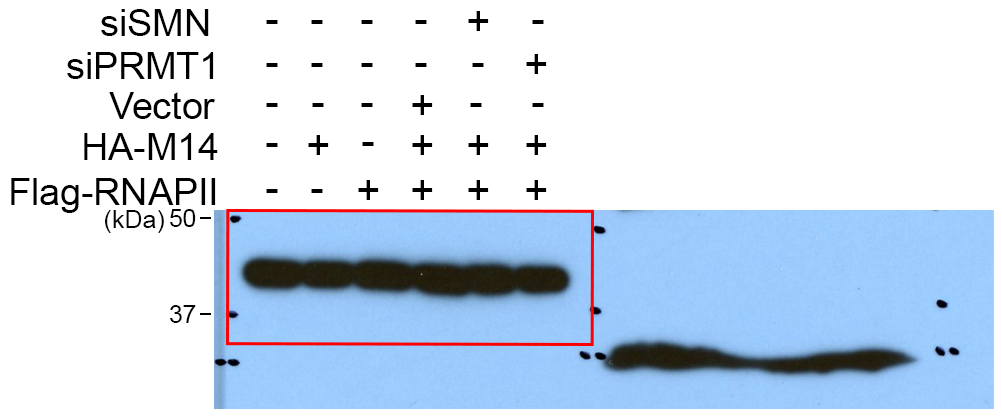

Supplement: Supplementary file 5 — Source data Fig. 3 [file 44319_2025_590_MOESM5_ESM.zip › Figure 3/3B/Western ACTIN.tif]

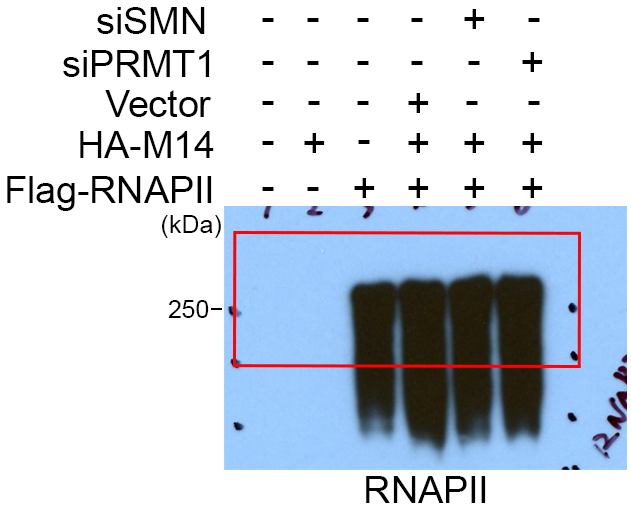

Supplement: Supplementary file 5 — Source data Fig. 3 [file 44319_2025_590_MOESM5_ESM.zip › Figure 3/3B/Western RNAP II (IP).tif]

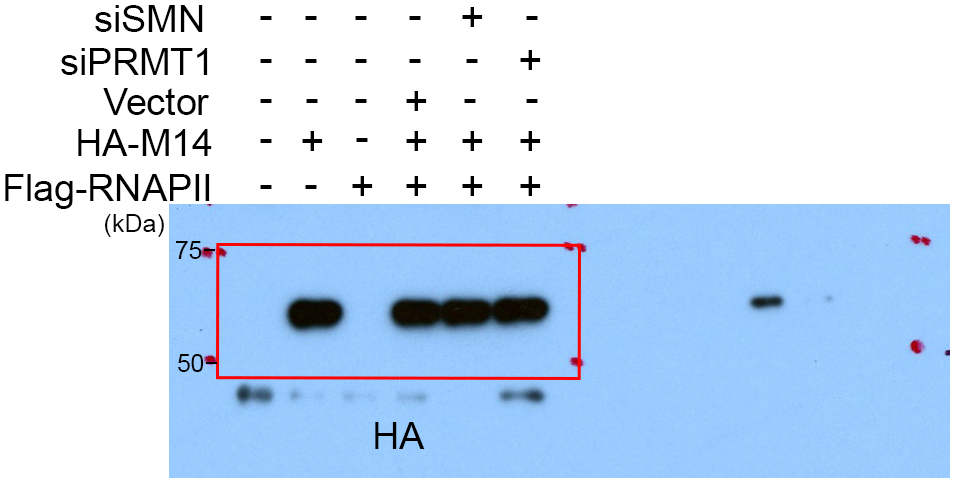

Supplement: Supplementary file 5 — Source data Fig. 3 [file 44319_2025_590_MOESM5_ESM.zip › Figure 3/3B/Western HA (Input).tif]

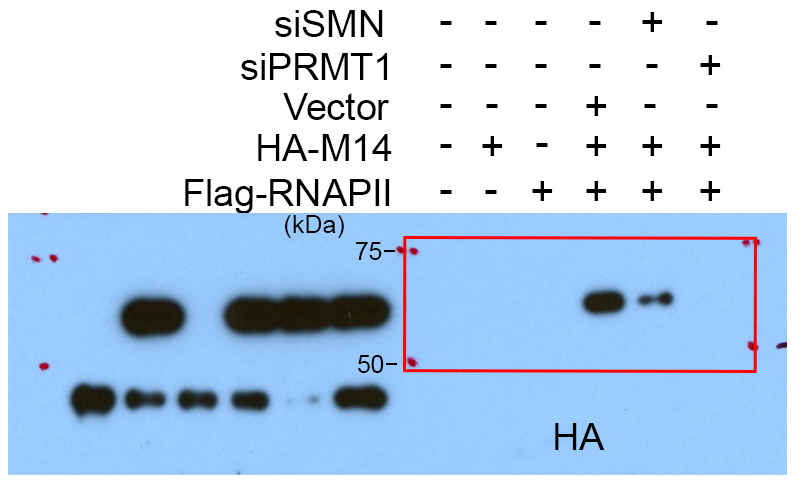

Supplement: Supplementary file 5 — Source data Fig. 3 [file 44319_2025_590_MOESM5_ESM.zip › Figure 3/3B/Western HA (IP).tif]

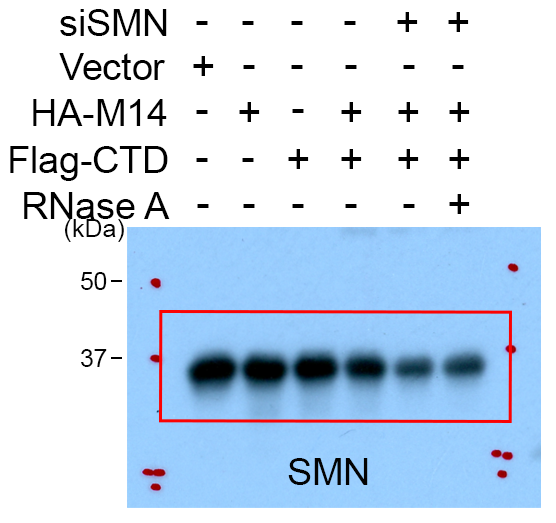

Supplement: Supplementary file 5 — Source data Fig. 3 [file 44319_2025_590_MOESM5_ESM.zip › Figure 3/3C/Western SMN.tif]

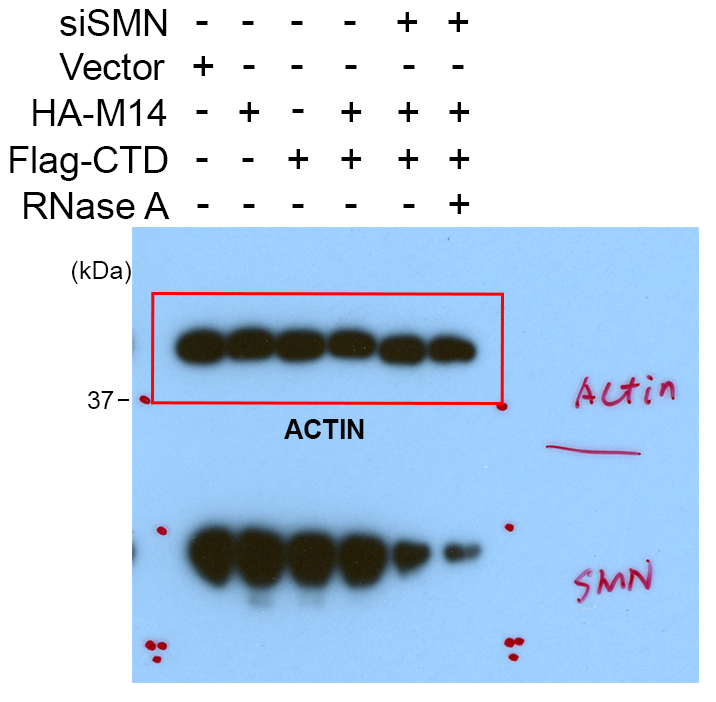

Supplement: Supplementary file 5 — Source data Fig. 3 [file 44319_2025_590_MOESM5_ESM.zip › Figure 3/3C/Western ACTIN.tif]

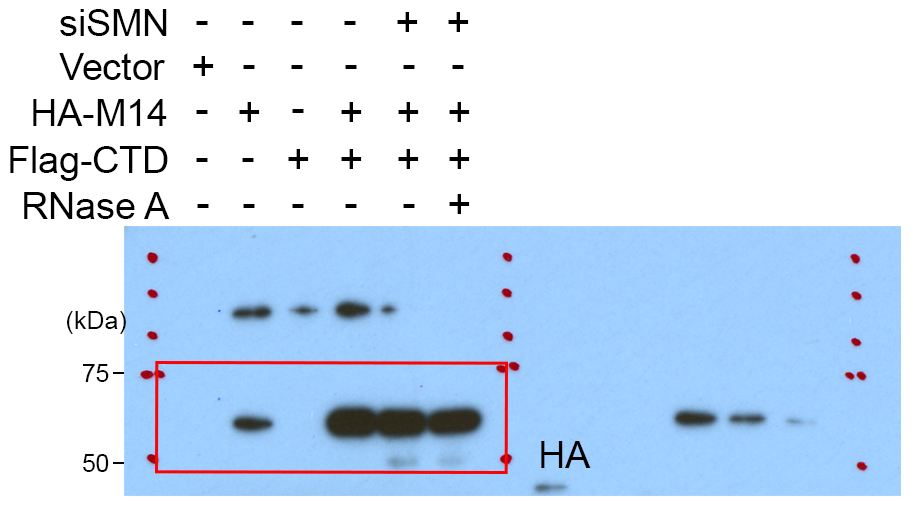

Supplement: Supplementary file 5 — Source data Fig. 3 [file 44319_2025_590_MOESM5_ESM.zip › Figure 3/3C/Western HA (Input).tif]

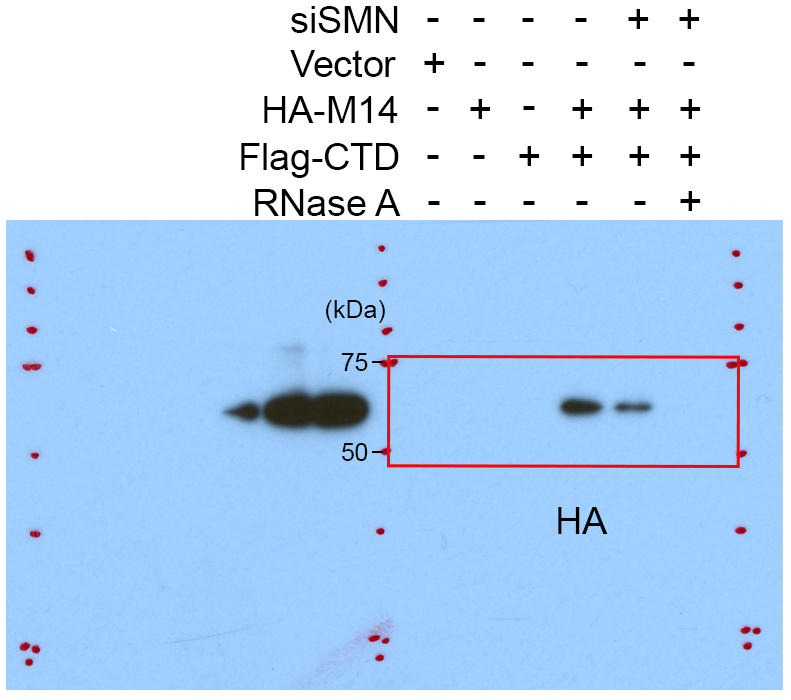

Supplement: Supplementary file 5 — Source data Fig. 3 [file 44319_2025_590_MOESM5_ESM.zip › Figure 3/3C/Western HA (IP).tif]

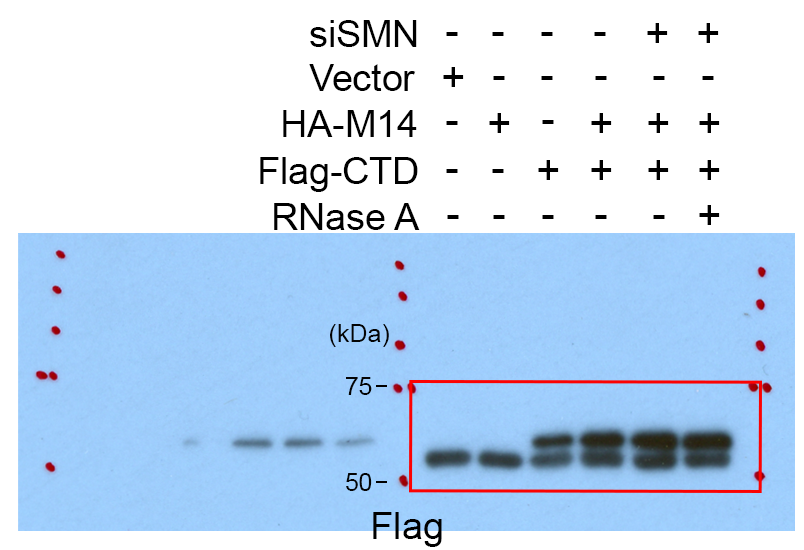

Supplement: Supplementary file 5 — Source data Fig. 3 [file 44319_2025_590_MOESM5_ESM.zip › Figure 3/3C/Western Flag (IP).tif]

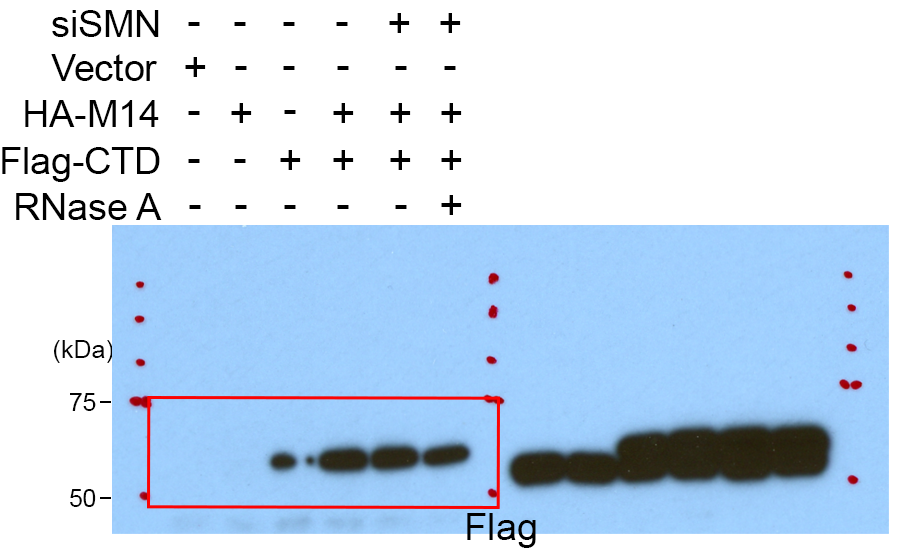

Supplement: Supplementary file 5 — Source data Fig. 3 [file 44319_2025_590_MOESM5_ESM.zip › Figure 3/3C/Western Flag (Input).tif]

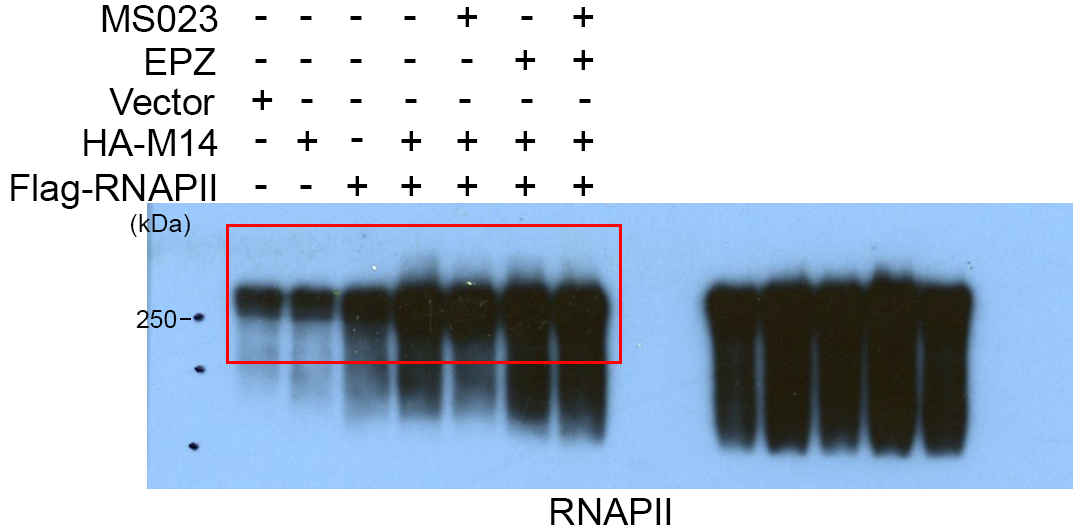

Supplement: Supplementary file 5 — Source data Fig. 3 [file 44319_2025_590_MOESM5_ESM.zip › Figure 3/3A/Western RNAPII (Input).tif]

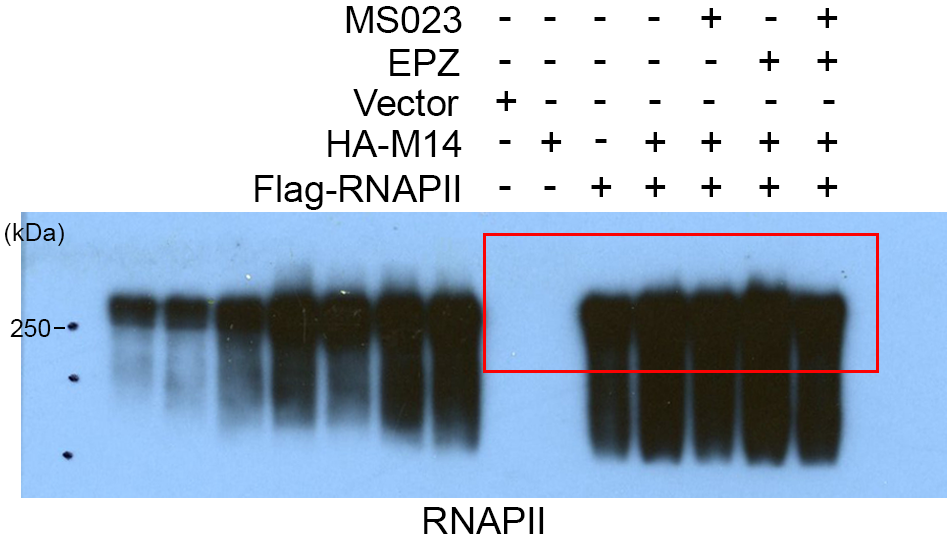

Supplement: Supplementary file 5 — Source data Fig. 3 [file 44319_2025_590_MOESM5_ESM.zip › Figure 3/3A/Western RNAPII (IP).tif]

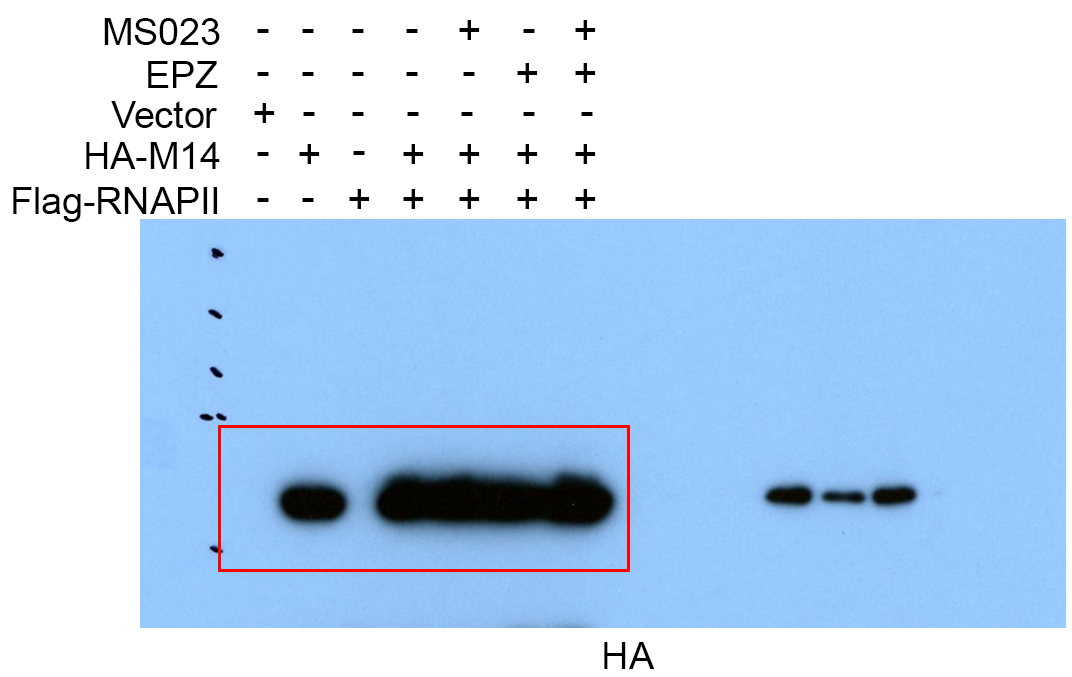

Supplement: Supplementary file 5 — Source data Fig. 3 [file 44319_2025_590_MOESM5_ESM.zip › Figure 3/3A/Western HA (Input).tif]

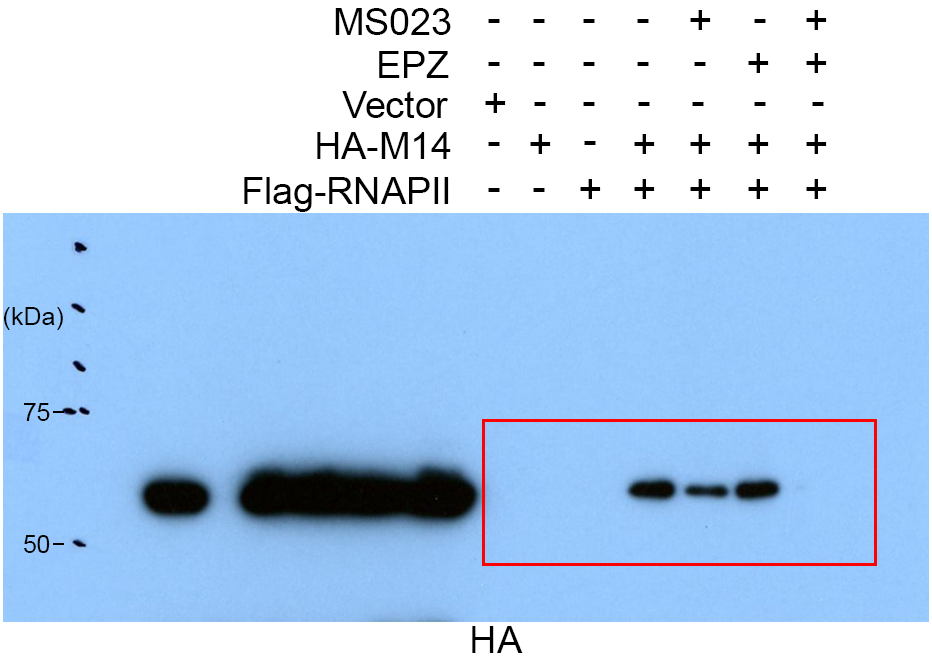

Supplement: Supplementary file 5 — Source data Fig. 3 [file 44319_2025_590_MOESM5_ESM.zip › Figure 3/3A/Western HA (IP).tif]

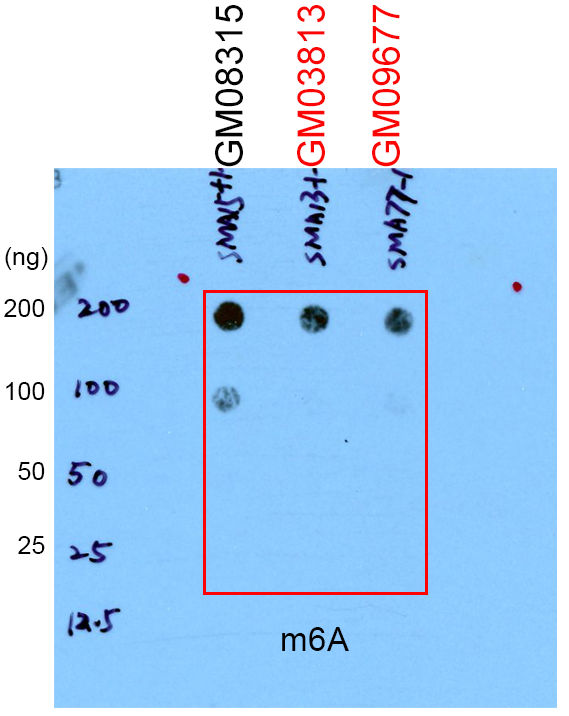

Supplement: Supplementary file 6 — Source data Fig. 4 [file 44319_2025_590_MOESM6_ESM.zip › Figure 4/4E/Dot blot m6A.tif]

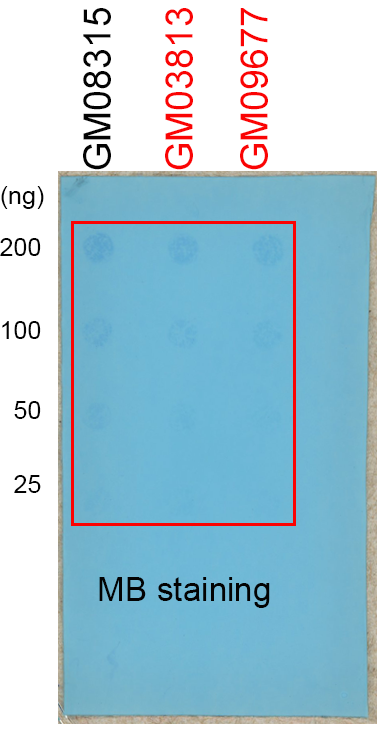

Supplement: Supplementary file 6 — Source data Fig. 4 [file 44319_2025_590_MOESM6_ESM.zip › Figure 4/4E/Dot blot MB staining.tif]

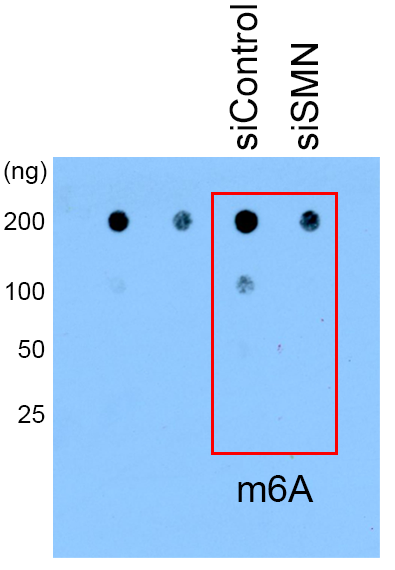

Supplement: Supplementary file 6 — Source data Fig. 4 [file 44319_2025_590_MOESM6_ESM.zip › Figure 4/4B/Dot blot m6A.tif]

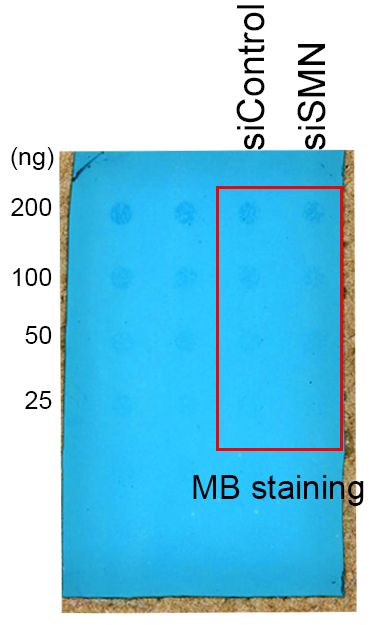

Supplement: Supplementary file 6 — Source data Fig. 4 [file 44319_2025_590_MOESM6_ESM.zip › Figure 4/4B/Dot blot MB staining.tif]

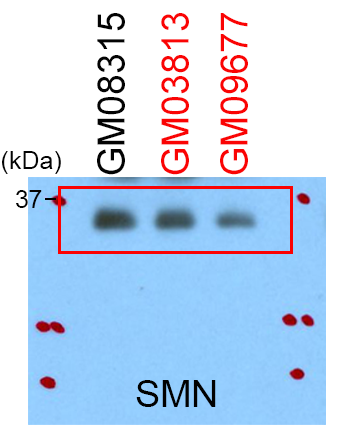

Supplement: Supplementary file 6 — Source data Fig. 4 [file 44319_2025_590_MOESM6_ESM.zip › Figure 4/4D/Western SMN.tif]

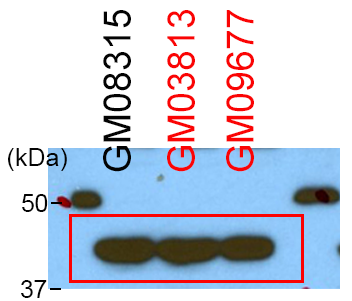

Supplement: Supplementary file 6 — Source data Fig. 4 [file 44319_2025_590_MOESM6_ESM.zip › Figure 4/4D/Western ACTIN.tif]

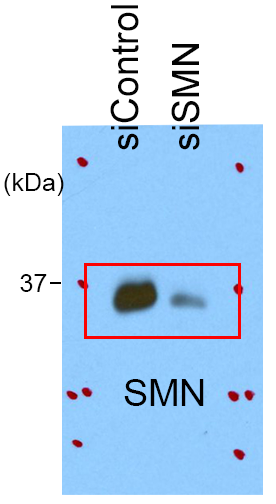

Supplement: Supplementary file 6 — Source data Fig. 4 [file 44319_2025_590_MOESM6_ESM.zip › Figure 4/4A/Western SMN.tif]

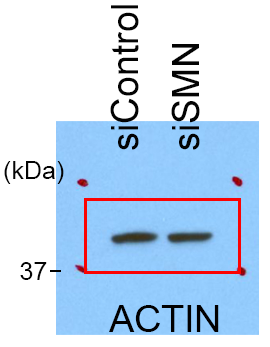

Supplement: Supplementary file 6 — Source data Fig. 4 [file 44319_2025_590_MOESM6_ESM.zip › Figure 4/4A/Western ACTIN.tif]

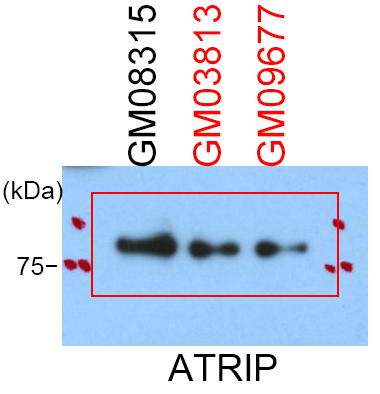

Supplement: Supplementary file 7 — Source data Fig. 5 [file 44319_2025_590_MOESM7_ESM.zip › Figure 5/5G/Western ATRIP.tif]

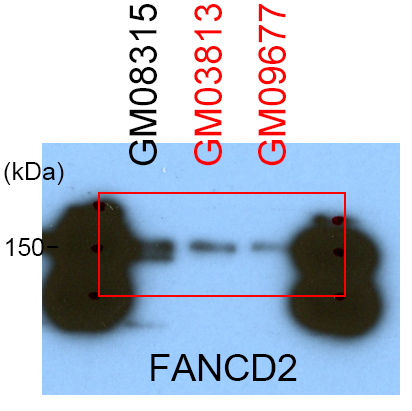

Supplement: Supplementary file 7 — Source data Fig. 5 [file 44319_2025_590_MOESM7_ESM.zip › Figure 5/5G/Western FANCD2.tif]

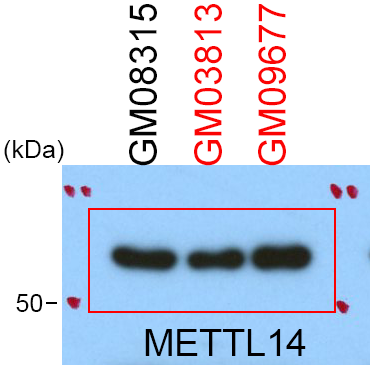

Supplement: Supplementary file 7 — Source data Fig. 5 [file 44319_2025_590_MOESM7_ESM.zip › Figure 5/5G/Western METTL14.tif]

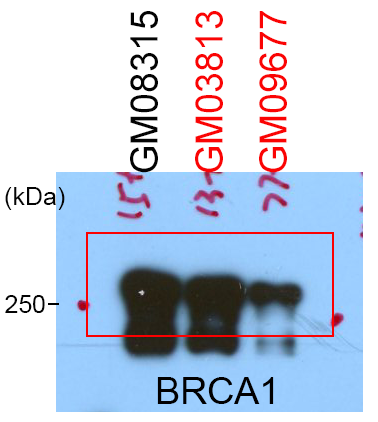

Supplement: Supplementary file 7 — Source data Fig. 5 [file 44319_2025_590_MOESM7_ESM.zip › Figure 5/5G/Western BRCA1.tif]

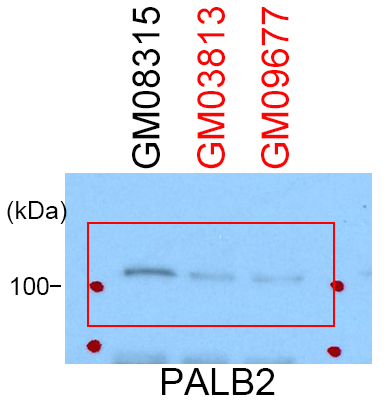

Supplement: Supplementary file 7 — Source data Fig. 5 [file 44319_2025_590_MOESM7_ESM.zip › Figure 5/5G/Western PALB2.tif]

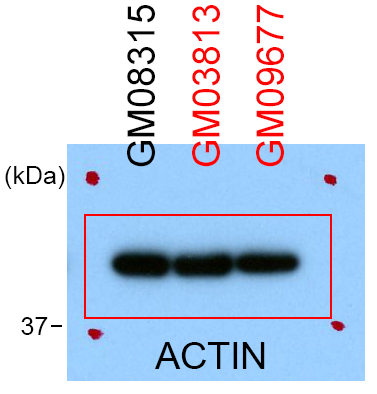

Supplement: Supplementary file 7 — Source data Fig. 5 [file 44319_2025_590_MOESM7_ESM.zip › Figure 5/5G/Western ACTIN.tif]

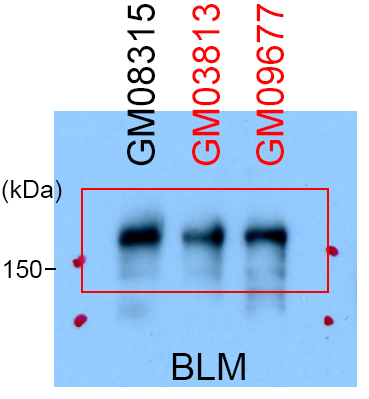

Supplement: Supplementary file 7 — Source data Fig. 5 [file 44319_2025_590_MOESM7_ESM.zip › Figure 5/5G/Western BLM.tif]

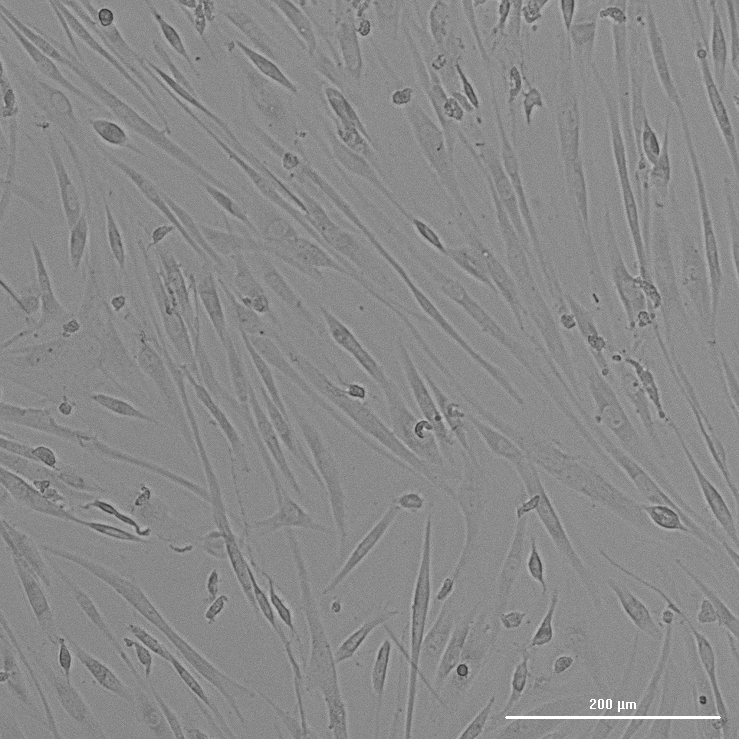

Supplement: Supplementary file 7 — Source data Fig. 5 [file 44319_2025_590_MOESM7_ESM.zip › Figure 5/5H/GM03813 MMC.png]

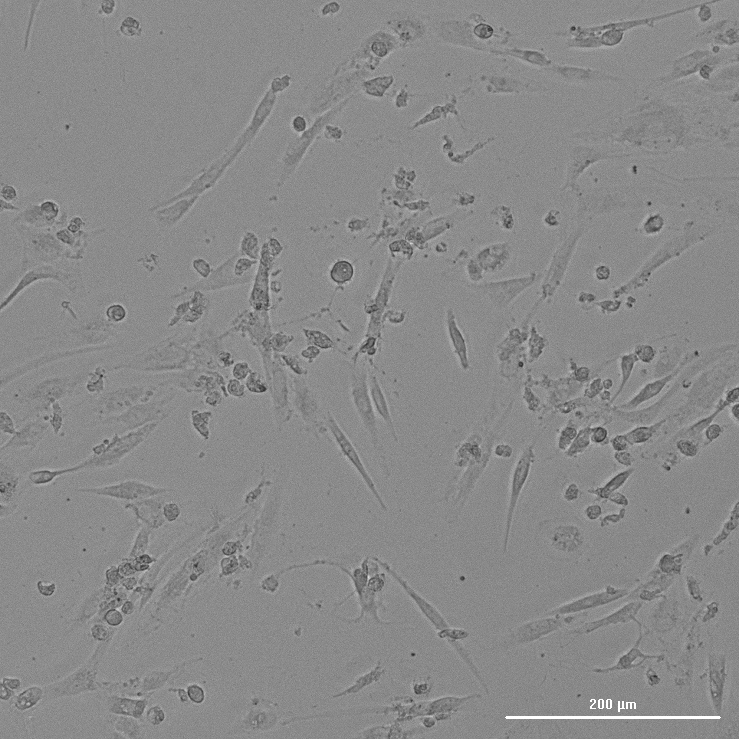

Supplement: Supplementary file 7 — Source data Fig. 5 [file 44319_2025_590_MOESM7_ESM.zip › Figure 5/5H/GM09677 MMC.png]

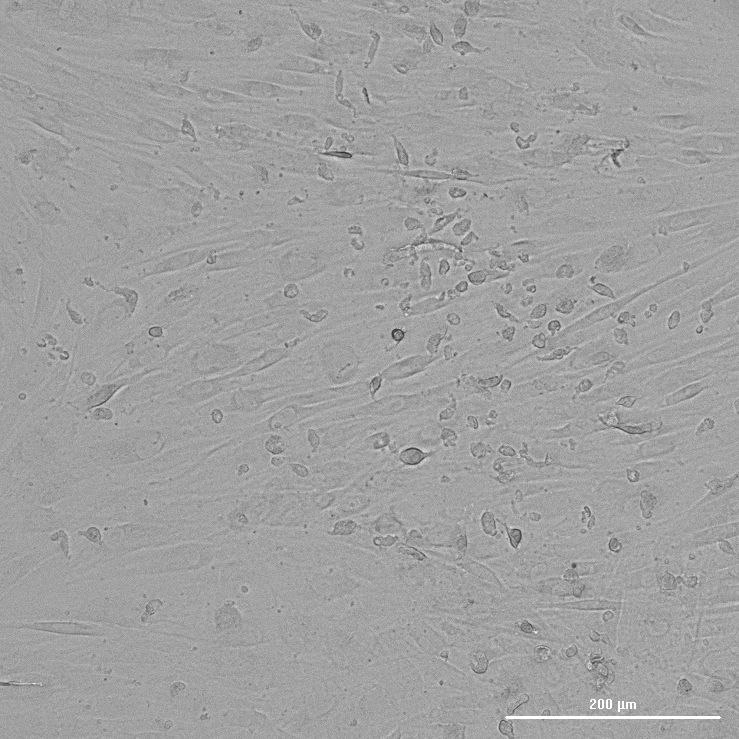

Supplement: Supplementary file 7 — Source data Fig. 5 [file 44319_2025_590_MOESM7_ESM.zip › Figure 5/5H/GM03813 Cisplatin.png]

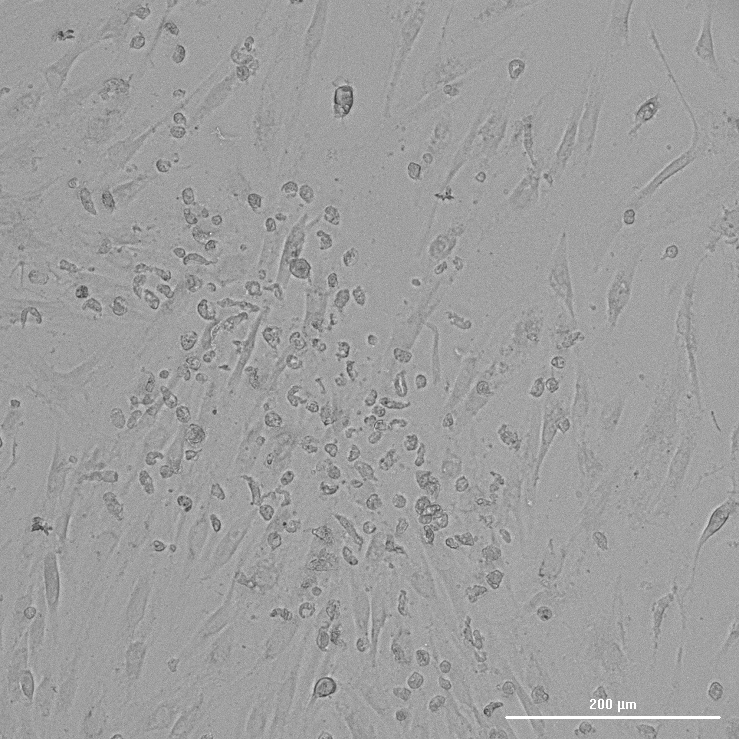

Supplement: Supplementary file 7 — Source data Fig. 5 [file 44319_2025_590_MOESM7_ESM.zip › Figure 5/5H/GM09677 Cisplatin.png]

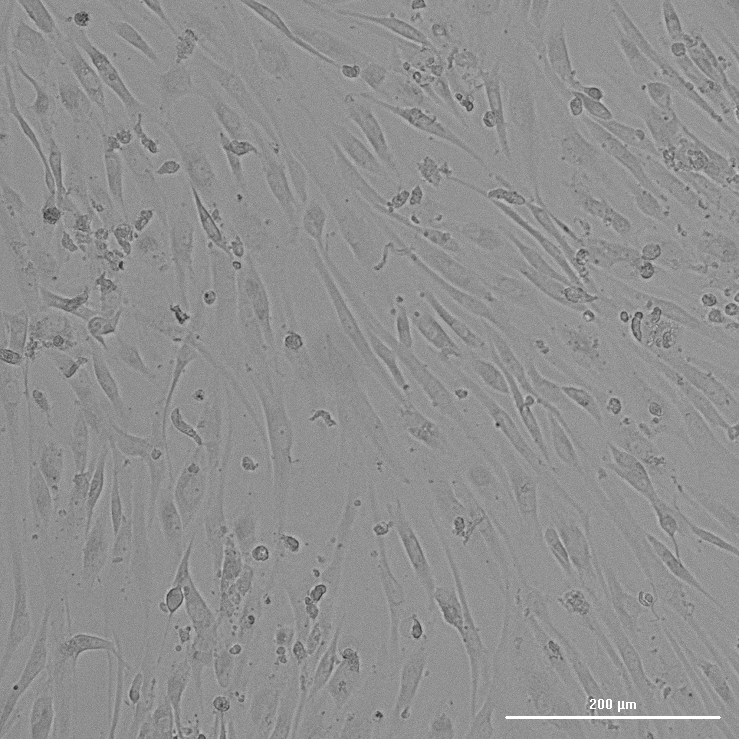

Supplement: Supplementary file 7 — Source data Fig. 5 [file 44319_2025_590_MOESM7_ESM.zip › Figure 5/5H/GM03815 MMC.png]

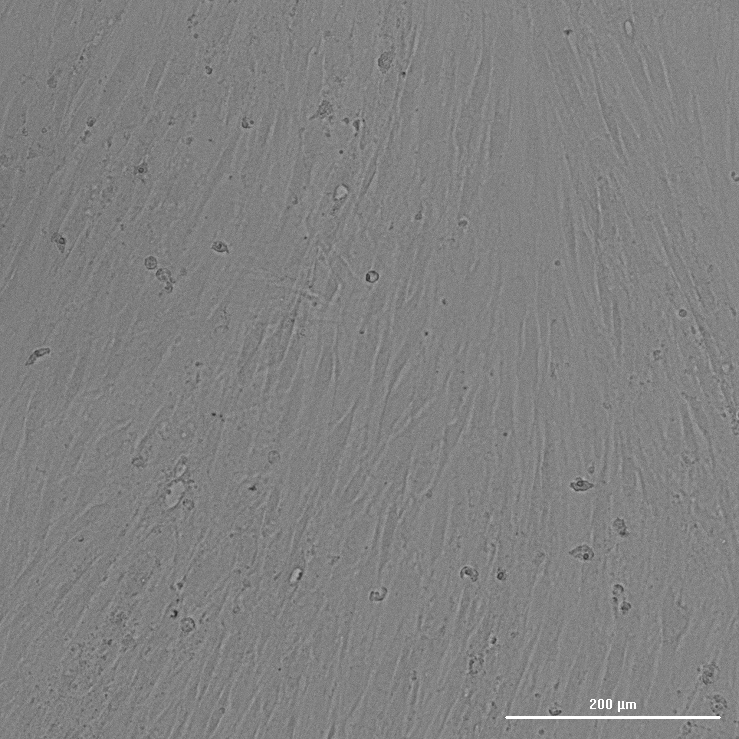

Supplement: Supplementary file 7 — Source data Fig. 5 [file 44319_2025_590_MOESM7_ESM.zip › Figure 5/5H/GM03813 DMSO.png]

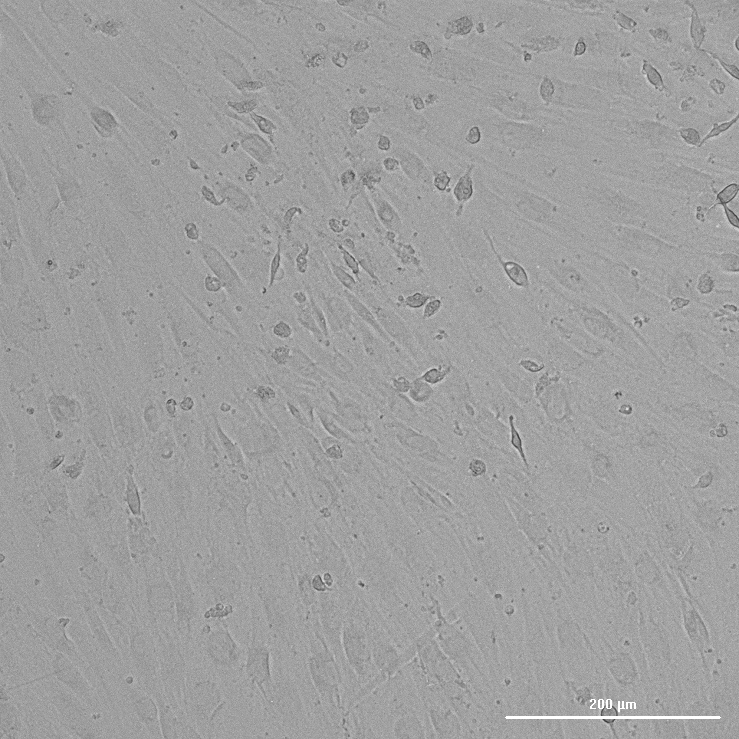

Supplement: Supplementary file 7 — Source data Fig. 5 [file 44319_2025_590_MOESM7_ESM.zip › Figure 5/5H/GM03815 Cisplatin.png]

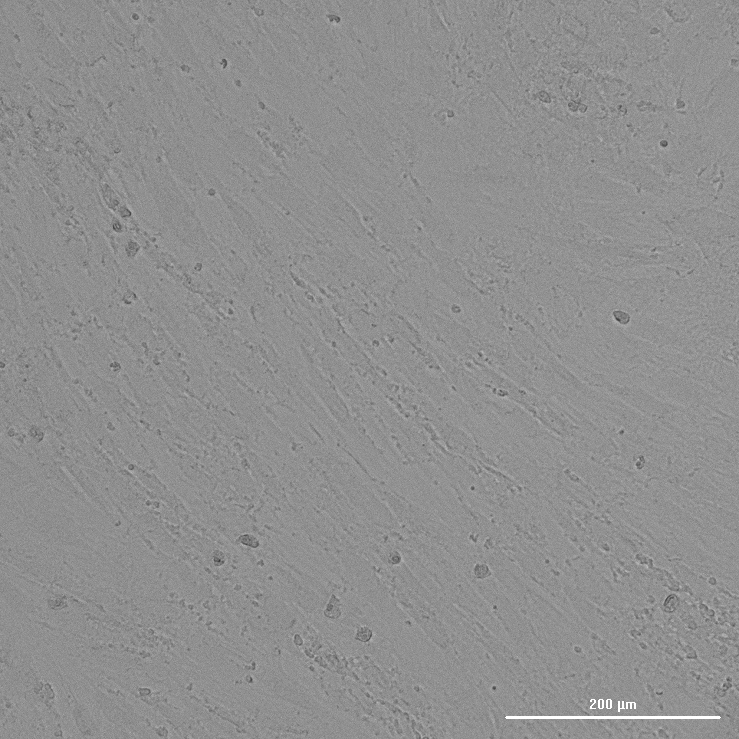

Supplement: Supplementary file 7 — Source data Fig. 5 [file 44319_2025_590_MOESM7_ESM.zip › Figure 5/5H/GM03815 DMSO.png]

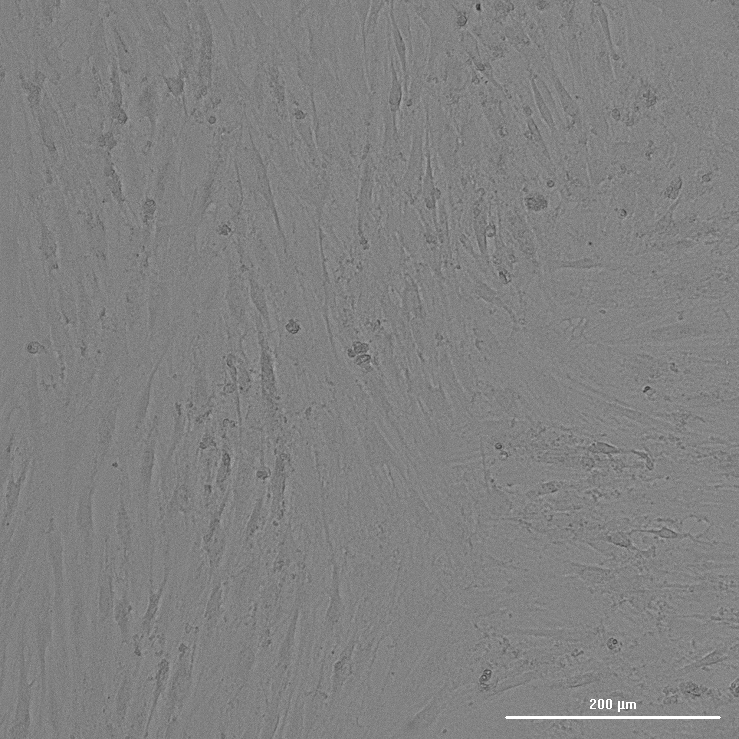

Supplement: Supplementary file 7 — Source data Fig. 5 [file 44319_2025_590_MOESM7_ESM.zip › Figure 5/5H/GM09677 DMSO.png]

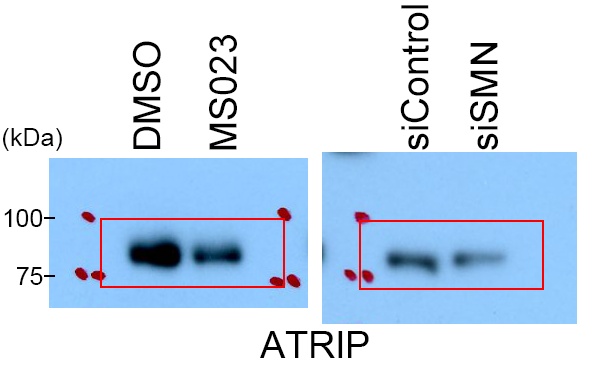

Supplement: Supplementary file 7 — Source data Fig. 5 [file 44319_2025_590_MOESM7_ESM.zip › Figure 5/5C/Western ATRIP.tif]

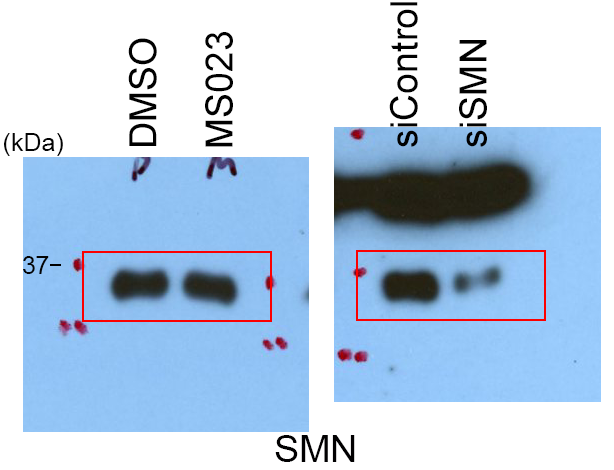

Supplement: Supplementary file 7 — Source data Fig. 5 [file 44319_2025_590_MOESM7_ESM.zip › Figure 5/5C/Western SMN.tif]

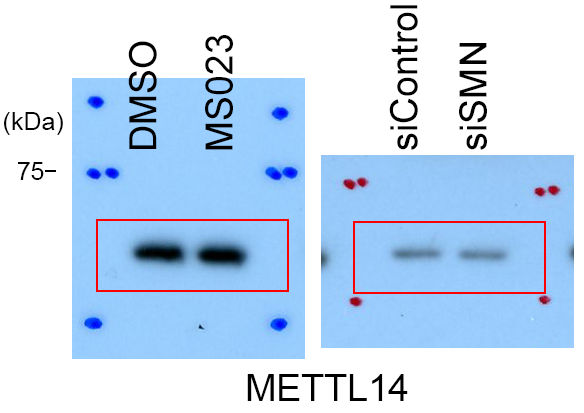

Supplement: Supplementary file 7 — Source data Fig. 5 [file 44319_2025_590_MOESM7_ESM.zip › Figure 5/5C/Western METTL14.tif]

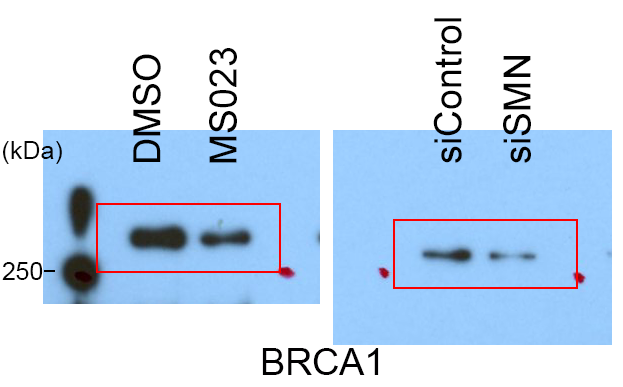

Supplement: Supplementary file 7 — Source data Fig. 5 [file 44319_2025_590_MOESM7_ESM.zip › Figure 5/5C/Western BRCA1.tif]

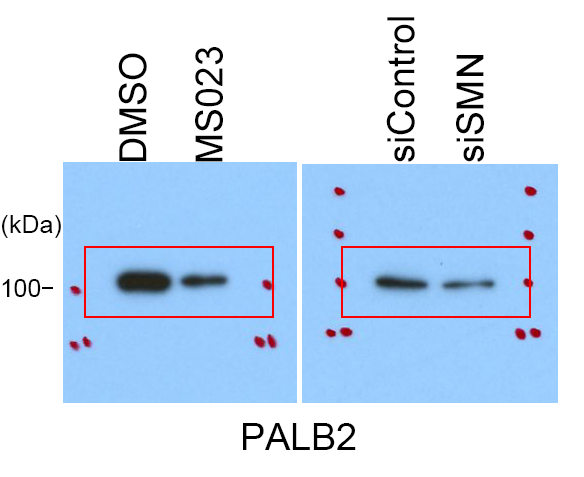

Supplement: Supplementary file 7 — Source data Fig. 5 [file 44319_2025_590_MOESM7_ESM.zip › Figure 5/5C/Western PALB2.tif]

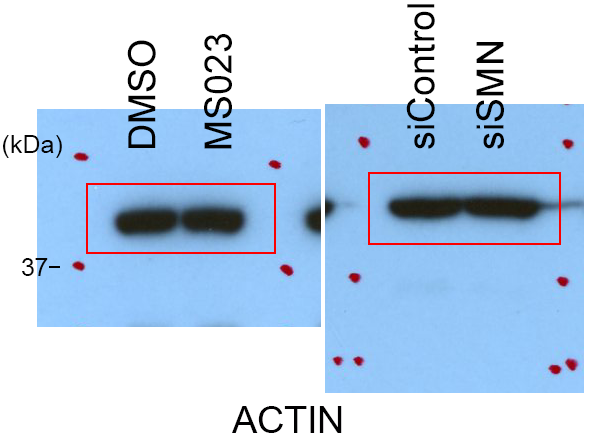

Supplement: Supplementary file 7 — Source data Fig. 5 [file 44319_2025_590_MOESM7_ESM.zip › Figure 5/5C/Western ACTIN.tif]

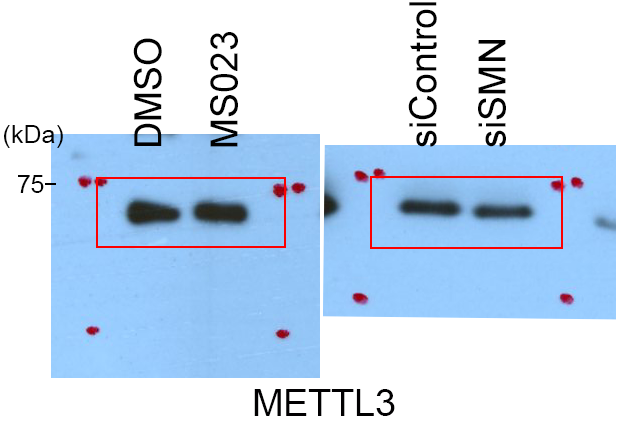

Supplement: Supplementary file 7 — Source data Fig. 5 [file 44319_2025_590_MOESM7_ESM.zip › Figure 5/5C/Western METTL3.tif]

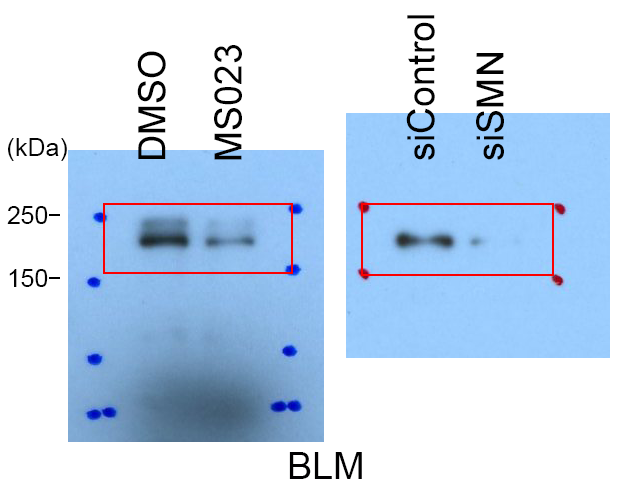

Supplement: Supplementary file 7 — Source data Fig. 5 [file 44319_2025_590_MOESM7_ESM.zip › Figure 5/5C/Western BLM.tif]

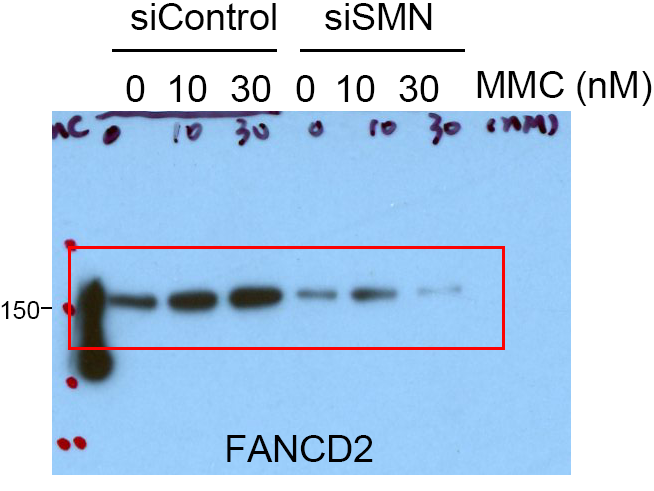

Supplement: Supplementary file 7 — Source data Fig. 5 [file 44319_2025_590_MOESM7_ESM.zip › Figure 5/5D/Western FANCD2.tif]

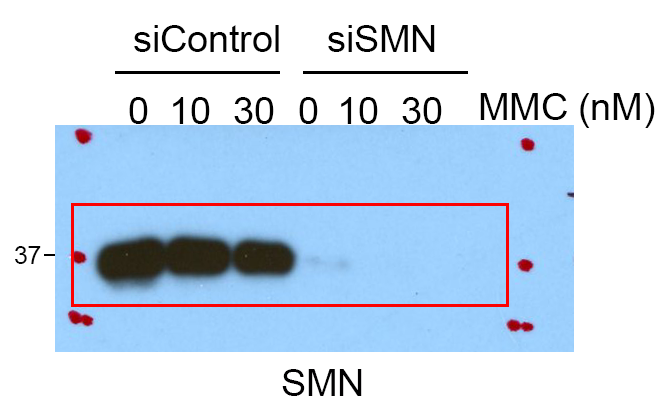

Supplement: Supplementary file 7 — Source data Fig. 5 [file 44319_2025_590_MOESM7_ESM.zip › Figure 5/5D/Western SMN.tif]

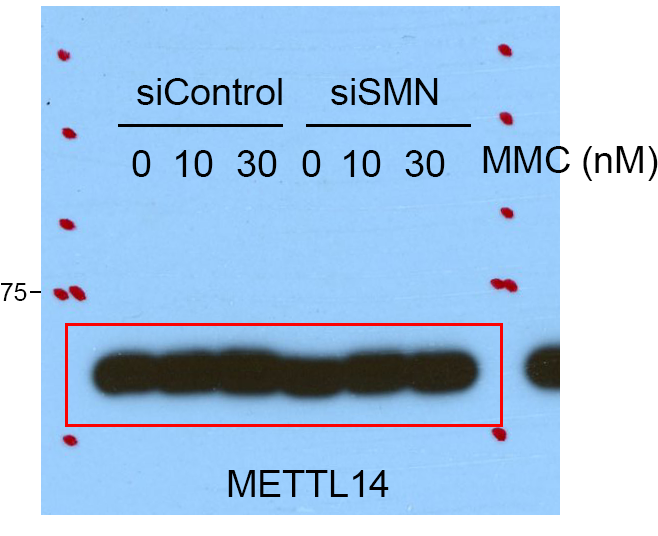

Supplement: Supplementary file 7 — Source data Fig. 5 [file 44319_2025_590_MOESM7_ESM.zip › Figure 5/5D/Western METTL14.tif]

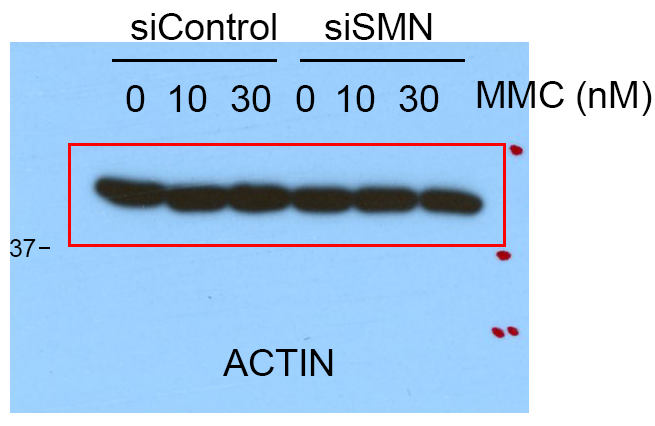

Supplement: Supplementary file 7 — Source data Fig. 5 [file 44319_2025_590_MOESM7_ESM.zip › Figure 5/5D/Western ACTIN.tif]

## Slide 1
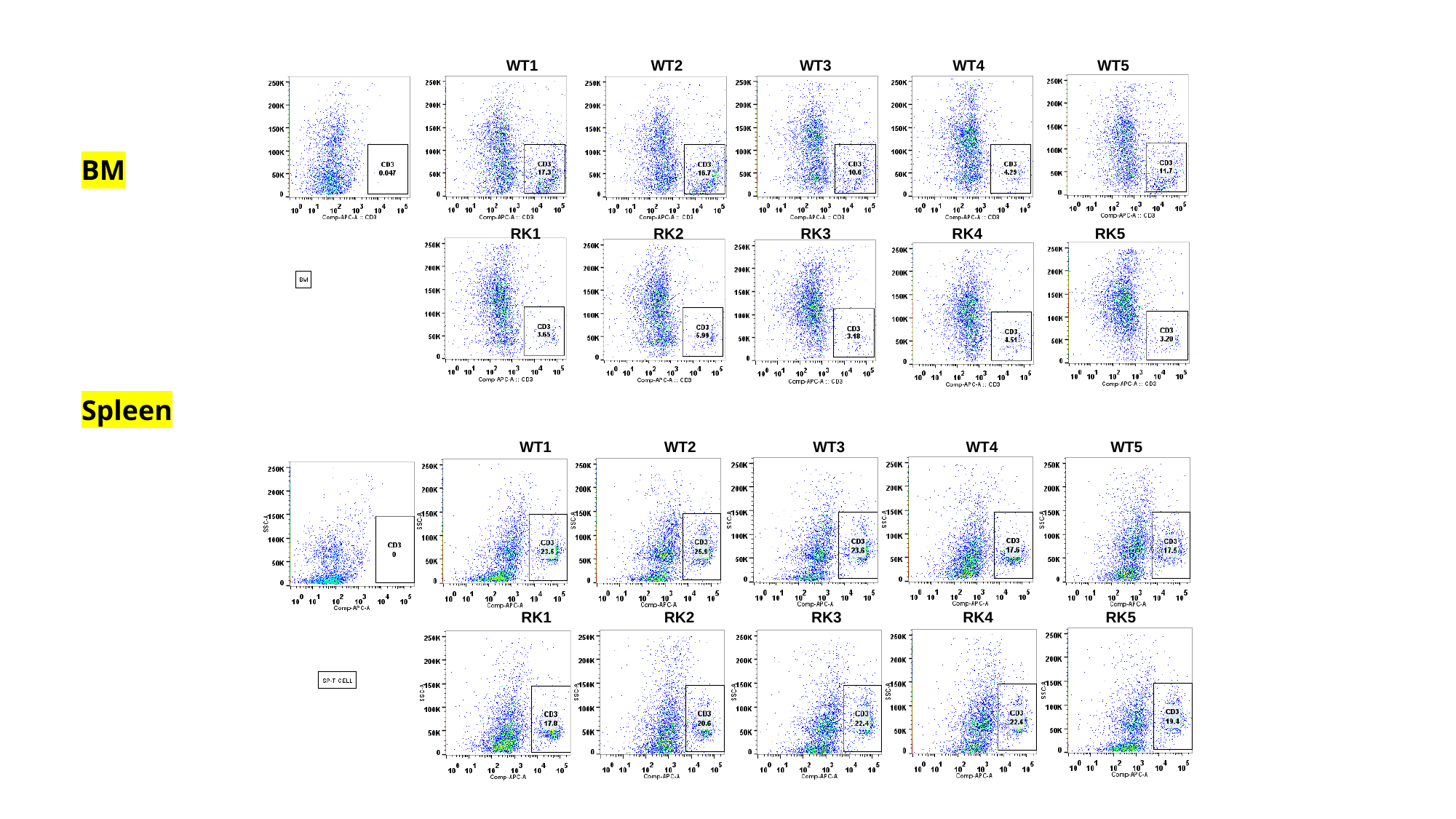

WT1 WT2 WT3 WT4 WT5
RK1 RK2 RK3 RK4 RK5
BM
Spleen
WT1 WT2 WT3 WT4 WT5
RK1 RK2 RK3 RK4 RK5

Supplement: Supplementary file 8 — Source data Fig. 6 [file 44319_2025_590_MOESM8_ESM.zip › Figure 6/6F/Results for 6F.pptx]

## Slide 1
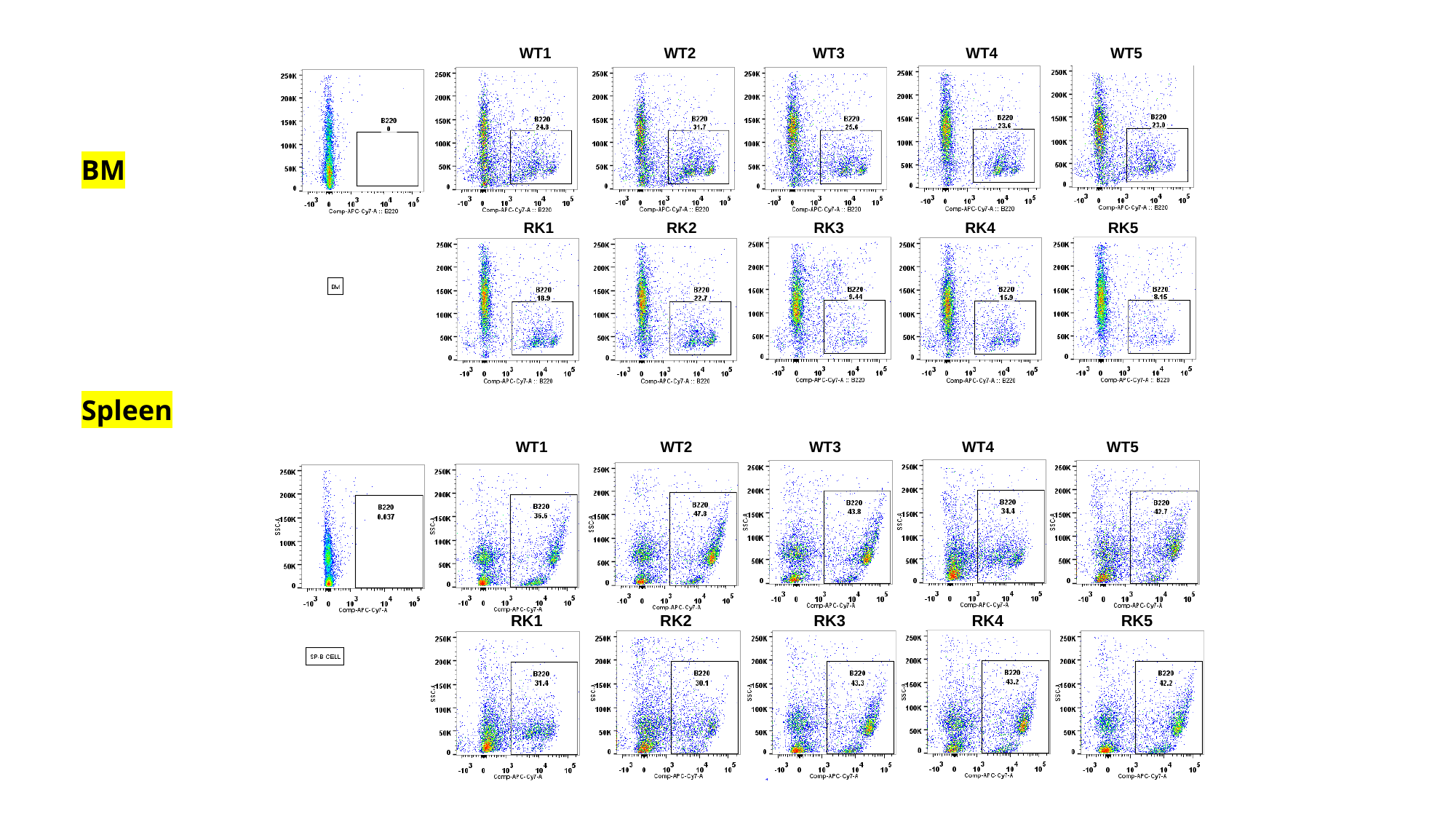

WT1 WT2 WT3 WT4 WT5
RK1 RK2 RK3 RK4 RK5
BM
Spleen
WT1 WT2 WT3 WT4 WT5
RK1 RK2 RK3 RK4 RK5

Supplement: Supplementary file 8 — Source data Fig. 6 [file 44319_2025_590_MOESM8_ESM.zip › Figure 6/6G/Results for 6G.pptx]

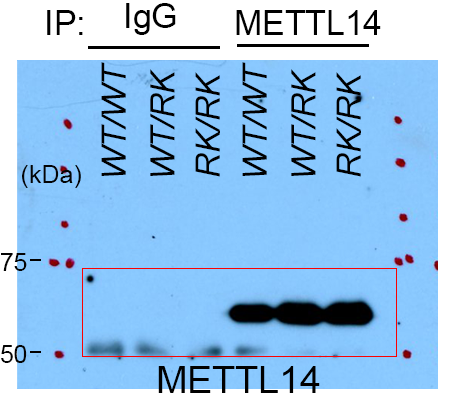

Supplement: Supplementary file 8 — Source data Fig. 6 [file 44319_2025_590_MOESM8_ESM.zip › Figure 6/6B/Western METTL14 (Spleen).tif]

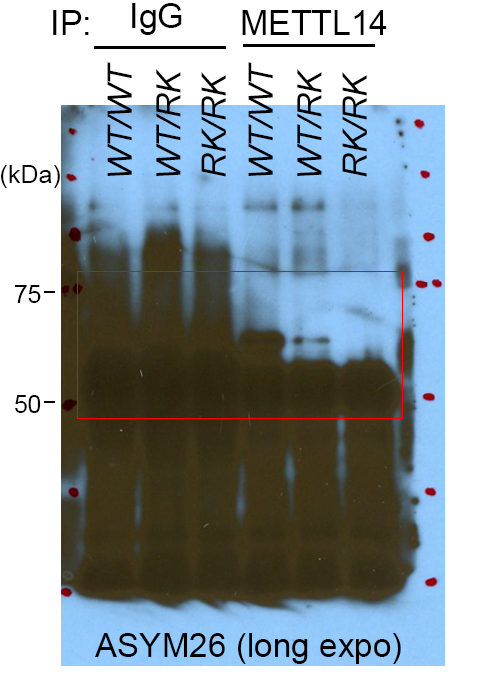

Supplement: Supplementary file 8 — Source data Fig. 6 [file 44319_2025_590_MOESM8_ESM.zip › Figure 6/6B/Western ASYM26 (long expo).tif]

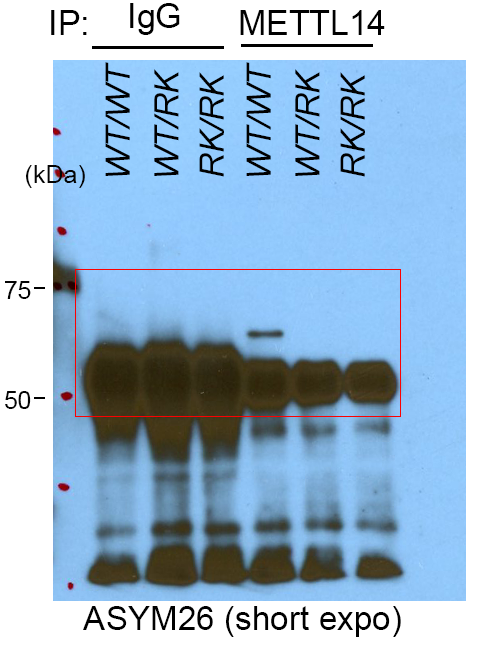

Supplement: Supplementary file 8 — Source data Fig. 6 [file 44319_2025_590_MOESM8_ESM.zip › Figure 6/6B/Western ASYM26 (short expo).tif]

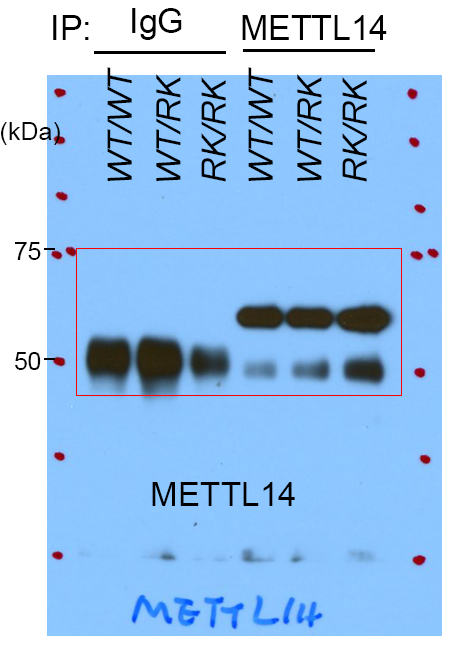

Supplement: Supplementary file 8 — Source data Fig. 6 [file 44319_2025_590_MOESM8_ESM.zip › Figure 6/6B/Western METTL14 (Thymus).tif]
