# Supplementary figures and images for: Arginine methylation-dependent METTL14-SMN interaction regulates RNA m6A homeostasis (part 2 of 2)
Source: EMBO Rep. 2025 Oct 6;26(22):5483–500. doi: 10.1038/s44319-025-00590-7 (PMC12635257; doi:10.1038/s44319-025-00590-7)

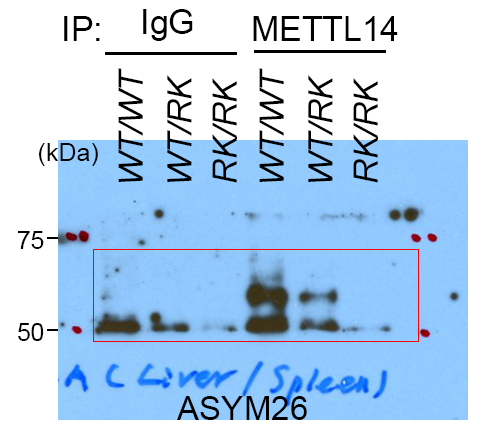

Supplement: Supplementary file 8 — Source data Fig. 6 [file 44319_2025_590_MOESM8_ESM.zip › Figure 6/6B/Western ASYM26 (Spleen).tif]

## Slide 1
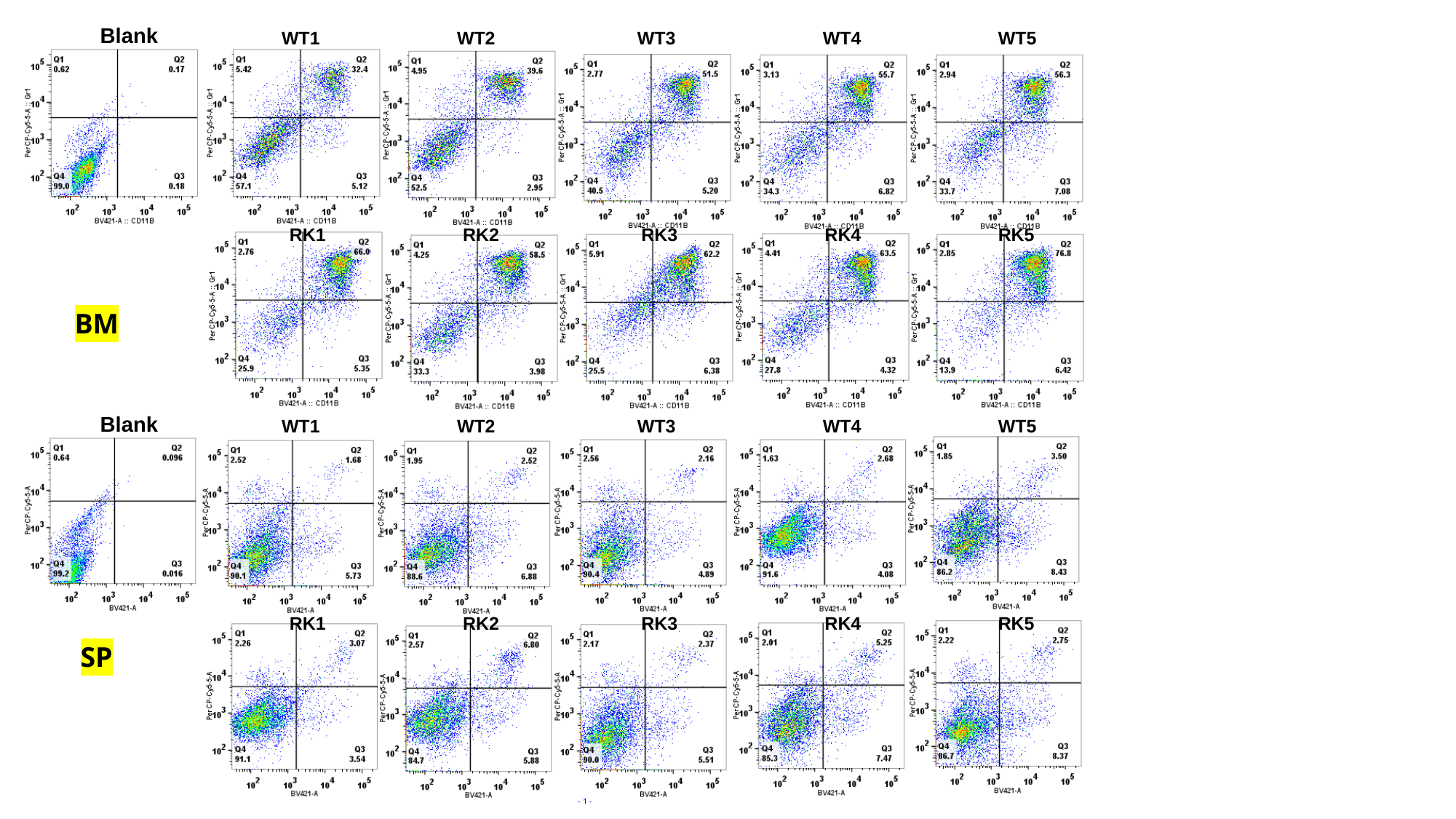

Blank
WT1 WT2 WT3 WT4 WT5
RK1 RK2 RK3 RK4 RK5
BM
Blank
WT1 WT2 WT3 WT4 WT5
RK1 RK2 RK3 RK4 RK5
SP

Supplement: Supplementary file 8 — Source data Fig. 6 [file 44319_2025_590_MOESM8_ESM.zip › Figure 6/6E/Results for 6E.pptx]

## Slide 1
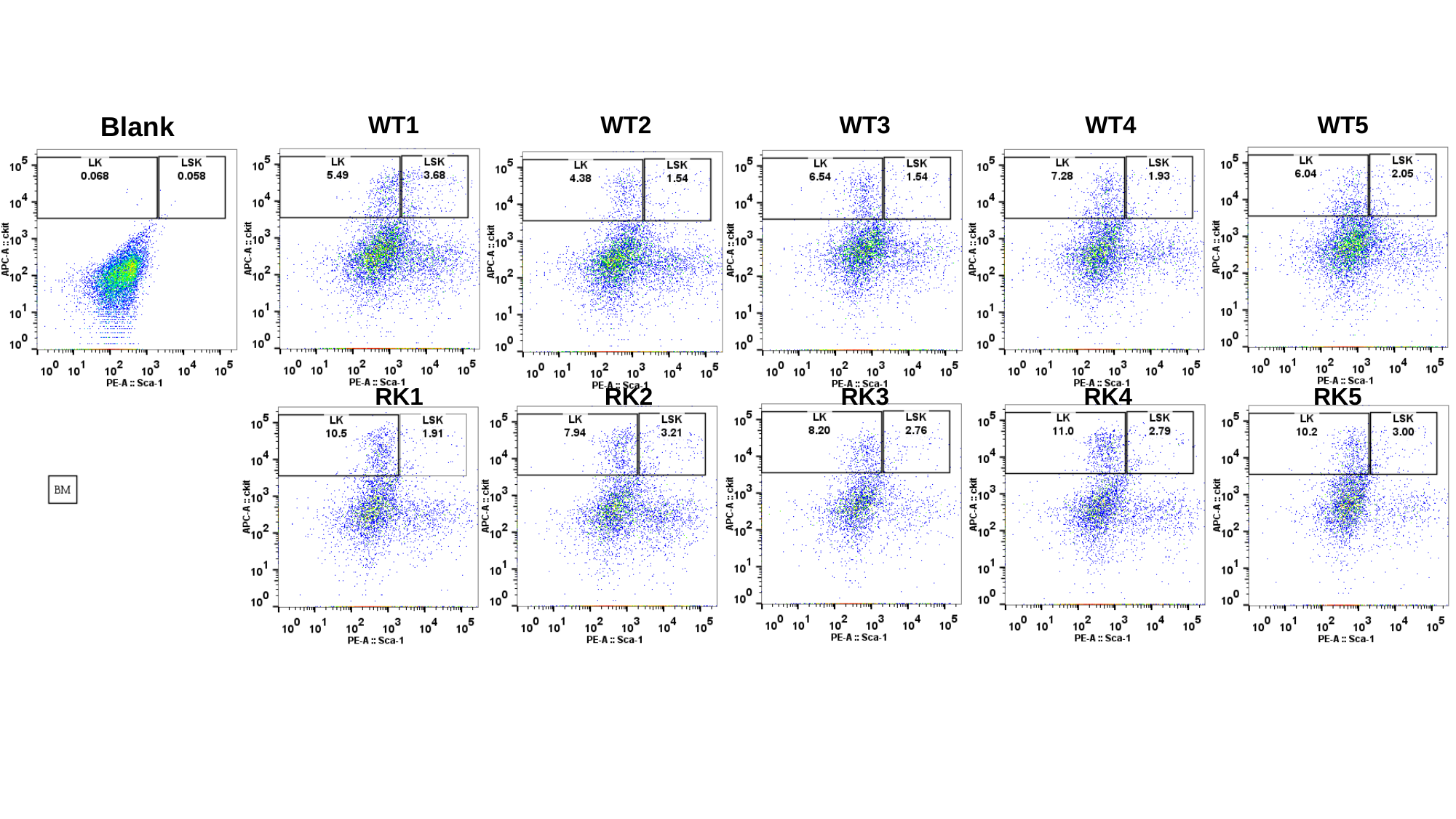

Blank
WT1 WT2 WT3 WT4 WT5
RK1 RK2 RK3 RK4 RK5

Supplement: Supplementary file 8 — Source data Fig. 6 [file 44319_2025_590_MOESM8_ESM.zip › Figure 6/6D/Results for 6D.pptx]

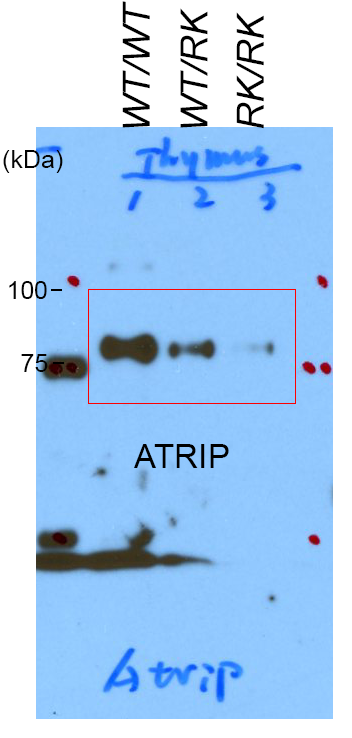

Supplement: Supplementary file 8 — Source data Fig. 6 [file 44319_2025_590_MOESM8_ESM.zip › Figure 6/6C/Western ATRIP.tif]

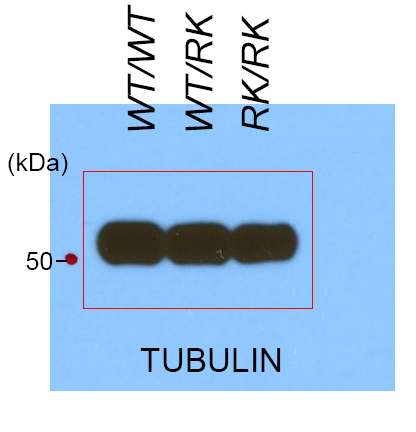

Supplement: Supplementary file 8 — Source data Fig. 6 [file 44319_2025_590_MOESM8_ESM.zip › Figure 6/6C/Western TUBULIN.tif]

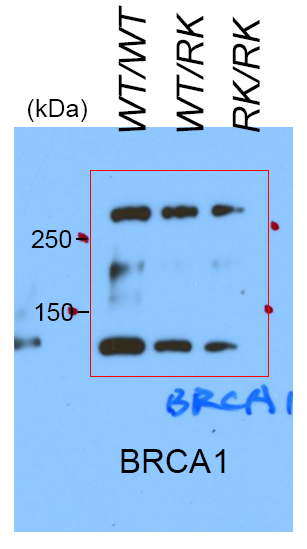

Supplement: Supplementary file 8 — Source data Fig. 6 [file 44319_2025_590_MOESM8_ESM.zip › Figure 6/6C/Western BRCA1.tif]

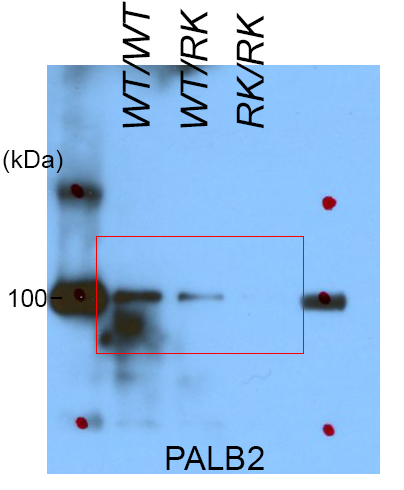

Supplement: Supplementary file 8 — Source data Fig. 6 [file 44319_2025_590_MOESM8_ESM.zip › Figure 6/6C/Western PALB2.tif]
